# CUL2-mediated clearance of misfolded TDP-43 is paradoxically affected by VHL in oligodendrocytes in ALS

**DOI:** 10.1038/srep19118

**Published:** 2016-01-11

**Authors:** Tsukasa Uchida, Yoshitaka Tamaki, Takashi Ayaki, Akemi Shodai, Seiji Kaji, Toshifumi Morimura, Yoshinori Banno, Kazuchika Nishitsuji, Naomi Sakashita, Takakuni Maki, Hirofumi Yamashita, Hidefumi Ito, Ryosuke Takahashi, Makoto Urushitani

**Affiliations:** 1 Department of Neurology, Kyoto University Graduate School of Medicine, 54 Shogoin-Kawahara-cho, Sakyo-ku, Kyoto, 606-8507 Japan; 2 Molecular Neuroscience Research Center, Shiga University of Medical Science, Seta-Tsukinowa-cho, Otsu, Shiga 520-2192, Japan; 3 Department of Molecular Pathology, Tokushima University, 2-24 Shinkuracho, Tokushima, Tokushima Prefecture 770-0855, Japan; 4 Department of Neurology, Wakayama Medical University, 811-1 Kimiidera, Wakayama, Wakayama Prefecture 641-8509, Japan.

## Abstract

The molecular machinery responsible for cytosolic accumulation of misfolded TDP-43 in amyotrophic lateral sclerosis (ALS) remains elusive. Here we identified a cullin-2 (CUL2) RING complex as a novel ubiquitin ligase for fragmented forms of TDP-43. The von Hippel Lindau protein (VHL), a substrate binding component of the complex, preferentially recognized misfolded TDP-43 at Glu246 in RNA-recognition motif 2. Recombinant full-length TDP-43 was structurally fragile and readily cleaved, suggesting that misfolded TDP-43 is cleared by VHL/CUL2 in a step-wise manner via fragmentation. Surprisingly, excess VHL stabilized and led to inclusion formation of TDP-43, as well as mutant SOD1, at the juxtanuclear protein quality control center. Moreover, TDP-43 knockdown elevated VHL expression in cultured cells, implying an aberrant interaction between VHL and mislocalized TDP-43 in ALS. Finally, cytoplasmic inclusions especially in oligodendrocytes in ALS spinal cords were immunoreactive to both phosphorylated TDP-43 and VHL. Thus, our results suggest that an imbalance in VHL and CUL2 may underlie oligodendrocyte dysfunction in ALS, and highlight CUL2 E3 ligase emerges as a novel therapeutic potential for ALS.

Amyotrophic lateral sclerosis (ALS) is a life-threatening neurodegenerative disorder that is characterized by progressive muscle atrophy and weakness. The majority of ALS cases are caused by abnormal conformation of ALS-linked proteins and by defective RNA handling, both of which induce various downstream cascades, including excitotoxicity, endoplasmic reticulum stress, mitochondrial dysfunction, glial dysfunction, proteasome impairment, unexpected secretion, and neuroinflammation^1^,^2^,^3^. Of considerable importance, TAR DNA-binding protein 43 kDa (TDP-43) was identified as a core component of ubiquitinated inclusions such as skein-like and round inclusions in sporadic ALS and ALS/frontotemporal lobar degeneration^4^,^5^. Epidemiological evidence of autosomal dominant inheritance of TDP-43 mutations in a subpopulation of familial ALS patients indicates the direct involvement of this protein in ALS pathogenesis^6^,^7^,^8^.

The exact mechanism of RNA mishandling in TDP-43 proteinopathy is unclear, but a loss of nuclear TDP-43 due to cytosolic mislocalization is considered a likely mechanism. Indeed, aggregate-prone TDP-43 proteins are readily mislocalized in the cytosol and sequester nuclear TDP-43^9^,^10^,^11^. We previously reported that a predominant RNA binding domain in TDP-43, RNA recognition motif (RRM) 1, was structurally and functionally vulnerable. Free state of Cys173 and Cys175 in RRM1 of TDP-43 preserves normal conformation, and their modification or substitution leads to misfolded TDP-43 reminiscent of that seen in ALS cytopathology, together with functional defects in RNA processing^11^. Interestingly, although we observed that wild-type (WT) and mislocalized TDP-43 with a mutant nucleus localizing signal (NLS) are comparatively pulled down with poly-ubiquitin in cell culture studies^12^, TDP-43 in ALS tissues or with cysteine substitution mutants in RRM1 is more heavily ubiquitinated than normally folded species^4^,^5^,^10^. These lines of evidence imply the presence of misfolded protein-specific handling machinery, which may be involved in ALS pathology. Parkin ubiquitinates WT and mutant TDP-43 to the same extent, and promotes aggregate formation in the cytosol^13^. The ubiquitin conjugating enzyme UBE2E3 also enhances ubiquitination of TDP-43, although the E3 ligase has not been identified^14^. As such, prior E3 candidates for TDP-43 may mediate physiological clearance of TDP-43, suggesting that other E3 ligases are involved in the pathological context.

Here, we found that the von Hippel Lindau (VHL)/cullin-2 (CUL2) E3 complex recognized and ubiquitinated misfolded TDP-43 and promoted clearance of fragmented forms of TDP-43. *VHL* is an anti-oncogenic gene that was originally identified as a cause of von Hippel Lindau disease, a dominantly inherited tumorigenic disorder in multiple organs. The VHL protein is a substrate binding protein in the CUL2 E3 complex and has been implicated in diverse pathways, including ubiquitination of hypoxia inducing factors (HIF)^15^,^16^ and an active form of protein kinase C^17^. However, no reports have shown the involvement of VHL/CUL2 in the clearance of TDP-43. Notably, we also found that when overexpressed, VHL augmented aggregate formation at the juxtanuclear protein quality control center (JUNQ), not only with TDP-43, but also familial ALS-linked mutants of superoxide dismutase 1(SOD1). Furthermore, we documented that glial cytoplasmic inclusions (GCI) in ALS patients were shown to comprise misfolded TDP-43 and VHL, which might underlie the oligodendrocyte dysfunction in ALS pathogenesis^18^,^19^,^20^.

## Results

### Interaction of TDP-43 with VHL/CUL2 E3 complexes

**Figure 1:**
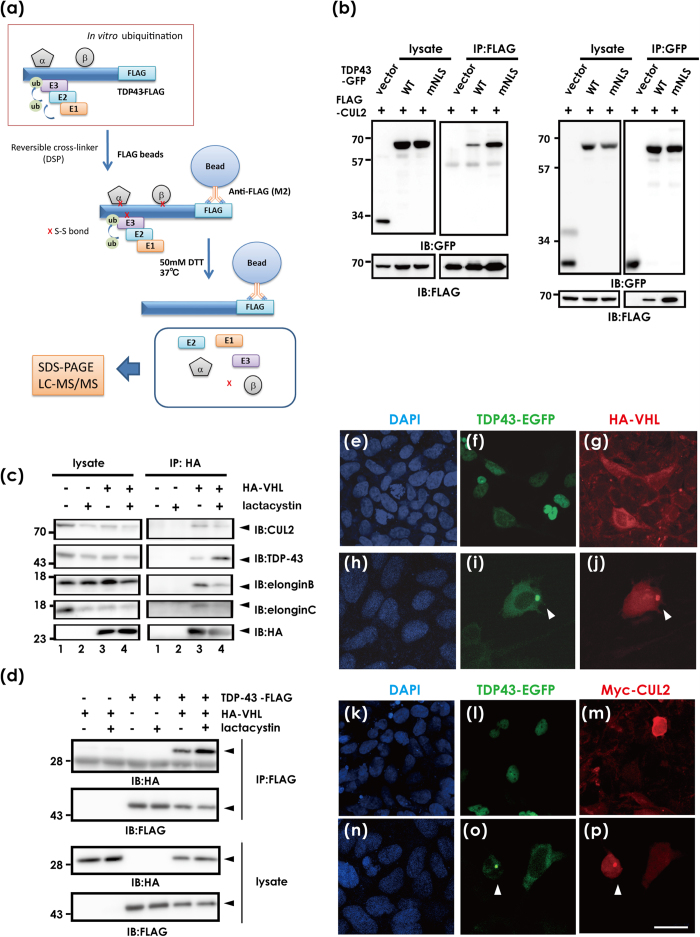
Interaction between TDP-43 and the VHL/CUL2 complex. (**a**) Schematic illustration of coupling of ReCLIP and *in vitro* ubiquitination to identify UPR-linked binding partners with TDP-43. First, recombinant TDP-43-FLAG proteins were incubated with S100T cell lysates potentially containing all components necessary for ubiquitination from HEK293A cells, followed by further reaction with the disulfide cross-linker DSP. After immunoprecipitation using anti-FLAG affinity gel, disulfide-linked proteins were released under mild reducing conditions. The eluates were analyzed by mass spectrometry. (**b**) *In vivo* pull-down assay of lysates from HeLa cells that were transfected with wild type (WT) TDP-43-GFP, TDP-43-GFP mutant with defective NLS (mNLS), and FLAG-CUL2, immunoprecipitated with anti-FLAG (left) or anti-GFP (right) affinity beads, and analyzed by Western blotting using anti-GFP or anti-FLAG antibodies, respectively. (**c**) Interaction of TDP-43 with VHL. Similarly, HEK293A cell lysates transfected with HA-VHL were immunoprecipitated with anti-HA affinity beads and immunoblotted for endogenous CUL2, elongin B, elongin C, and TDP-43. (**d**) The interaction between overexpressed TDP-43 and VHL was also shown by immunodetection of HA-VHL with immunoprecipitated TDP-43-FLAG. Lactacystin promoted binding of HA-VHL and TDP-43-FLAG. (**e–p**) Confocal laser micrographs of HEK293A cells expressing TDP-43-EGFP (as green) and HA-VHL (a–g, h–j) or Myc-CUL2 (k–m, n–p) 48 h after transfection. DAPI was used for counterstaining (blue). Occasional cytosolic inclusion of overexpressed WT TDP-43 colocalized with VHL (h–j) or CUL2 (n–p). Scale bar, 50 μm.

Because of the potential failure to seize labile protein complexes in specific conditions such as ubiquitination, we integrated *in vitro* ubiquitination and a reversible covalent immunoprecipitation assay^21^, in which recombinant human TDP-43-FLAG proteins were incubated in a ubiquitination reaction solution containing detergent-soluble S100 lysates from HEK293A cells, and the reaction mixtures were incubated with disulfide cross-linker, and then the immunoprecipitates with FLAG affinity beads were eluted under mild reducing condition (Fig. 1a). As a cross-linker, we used Dithiobis[succinimidyl propionate] (DSP), which has amine-reactive N-hydroxysuccinimide (NHS) esters at both ends of a cleavable, 8-atom (12.0 angstrom) spacer arm. Of the potential candidates that may interact with TDP-43 (Supplementary Table S1), we selected CUL2, a possible component of the ubiquitin ligase E3 complex. We first investigated the interaction between TDP-43 and CUL2 (Fig. 1b) or the substrate-binding protein VHL (Fig. 1c,d) with an immunoprecipitation assay of co-transfected HeLa or HEK293A cell lysates. We observed a trend in which the cytosolic TDP-43 with a mutant NLS interacted more with CUL2 than WT TDP-43 (Fig. 1b). Other components in the CUL2 E3 complex, including elongin B and C, were also contained together with hemagglutinin (HA)-VHL and endogenous TDP-43 (Fig. 1c). Proteasome inhibition by lactacystin promoted the interaction between TDP-43 and VHL (Fig. 1c,d), suggesting degradation of these proteins by proteasomes. VHL and CUL2 are cytosolic proteins as shown in transfected cells (Fig. 1g,m), whereas WT TDP-43 is present in the nucleus (Fig. 1f,l). However, overexpression of WT TDP-43 induced occasional cytosolic inclusions, which were colocalized with overexpressed VHL (Fig. 1i,j) or CUL2 (Fig. 1o,p), as well as with endogenous VHL (Supplementary Fig. S1). WT and several familial ALS-linked TDP-43 mutants showed comparable affinity for VHL (Supplementary Fig. S2).

### VHL preferentially recognizes misfolded forms of TDP-43

**Figure 2:**
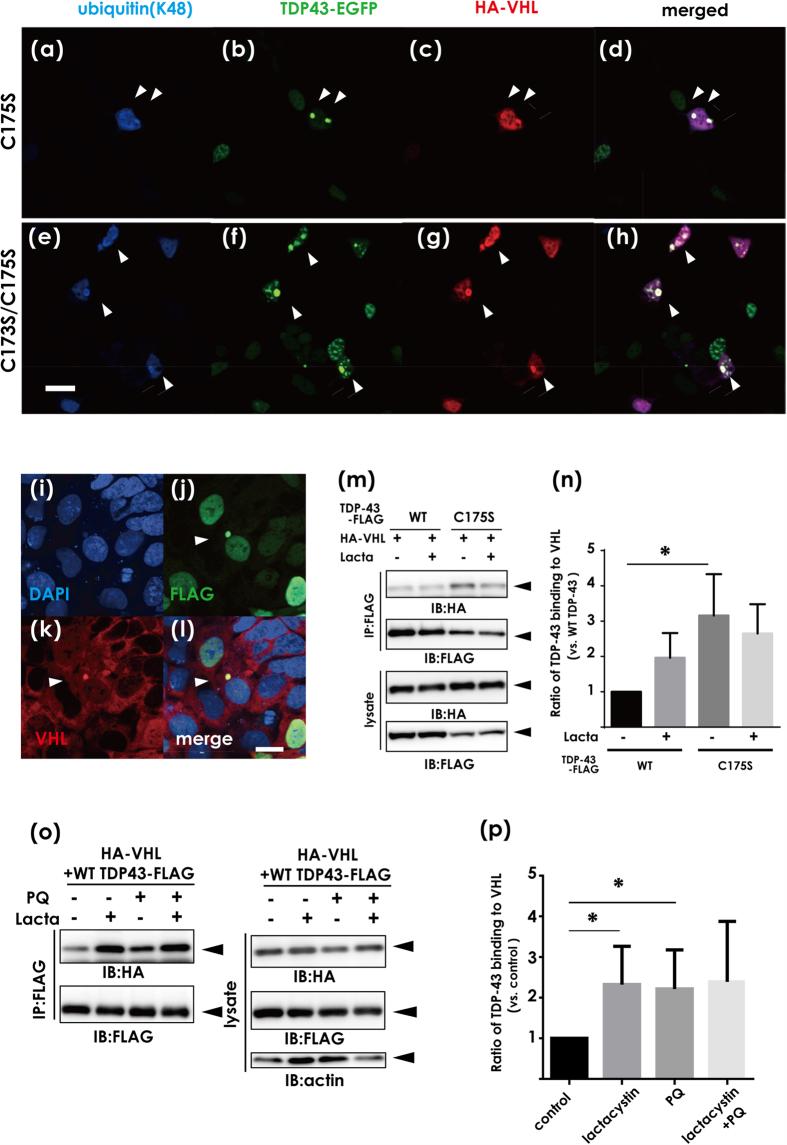
Effects of TDP-43 conformation on the interaction with the VHL/CUL2 complex. (**a–h**) Confocal laser micrographs of HEK293A cells expressing HA-VHL and aggregation-prone mutants (C173S and/or C175S). (**a–d**) for C175S and (**e–h**) for C173S/C175S. Arrowheads indicate co-localization of TDP-43 aggregates of ubiquitin and VHL. Scale bar, 50 μm. (**i–l**) Confocal laser micrographs showing that TDP-43 aggregates colocalize with endogenous VHL in HEK293A cells. HEK293A cells were fixed and assayed by immunofluorescence with antibodies against FLAG (green) and VHL (red). Scale bar, 20 μm. (**m,n**) The lysates from HEK293A cells were transfected with TDP-43-FLAG (WT or C175S) and HA-VHL, and the cell lysates were immunoprecipitated with anti-FLAG and analyzed by Western blotting using anti-HA or anti-FLAG antibodies (**m**), and by the densitometric analysis (**n**). Aggregation-prone mutants showed a significantly higher affinity with VHL than WT. Differences were evaluated by one-way ANOVA (mean ± SD from triplicates; *p < 0.05 versus WT). (**o**) Western blotting showing the effect of oxidative stress induced by paraquat on the binding affinity of TDP-43 to VHL. HEK293A cells transfected with HA-VHL and WT TDP-43-FLAG were treated with lactacystin (10 μM, 8 h) and/or paraquat (PQ, 0.4 mM, 8 h). (**p**) Bands detected by the anti-HA antibody in (**n**) were assessed by densitometry. Each data point was obtained by normalization to TDP-43 and is expressed as the average of three independent experiments. Differences were evaluated by one-way ANOVA (mean ± SD from triplicates; *p < 0.05 versus control).

Based on the finding that VHL and CUL2 colocalize with cytosolic inclusions of TDP-43, we investigated whether VHL/CUL2 recognizes misfolded forms of TDP-43 more than normally folded TDP-43. We previously reported that the conformation of TDP-43 is tightly regulated by two cysteine residues in RRM1, the substitution of which (C173S and/or C175S) leads to formation of aggregate-prone TDP-43 that shares diverse features of pathogenic inclusions in ALS^11^. Confocal laser microscope analysis of HEK293A cells overexpressing VHL and TDP-43 with cysteine substitution(s) to serine (C/S) showed that the majority of TDP-43 inclusions were immunopositive for VHL, polyubiquitin at K48 (Fig. 2a–d for C175S, e-h for C173S/C175S), and endogenous VHL (Fig. 2i–l, Supplementary Figure S1, i-p). An immunoprecipitation study also showed a significantly higher affinity of VHL for the C175S mutant than WT in co-transfected cell lysates (Fig. 2m,n). Moreover, treatment with paraquat, which induces stress granules^22^, revealed that more VHL was pulled down with TDP-43 under stress conditions (Fig. 2o,p). Similar to VHL, CUL2 was present within the cytosolic inclusions comprising aggregate-prone C/S mutant TDP-43 in HeLa cells (Supplementary Fig. S4, i-l).

### VHL recognizes E246 in the RRM2 domain, a potential epitope for misfolded TDP-43

**Figure 3:**
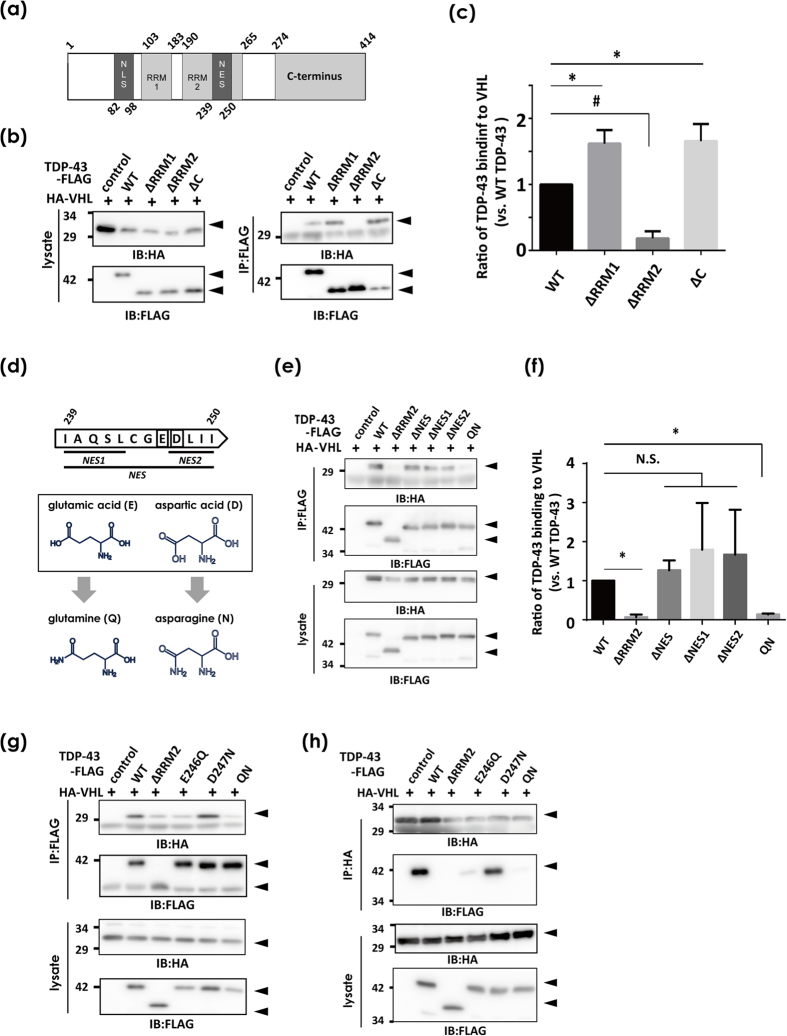
VHL recognition site in TDP-43. *In vivo* pull-down assay of HEK293A cells transfected with HA-VHL and WT TDP-43-FLAG devoid of various domains. (**a**) Schematic showing of the domain profiles of FL TDP-43. (**b,c**) RRM2 domain is required for the interaction between TDP-43 and VHL. Total lysates in co-IP buffer were immunoprecipitated with anti-FLAG affinity beads to pull-down TDP-43-FLAG devoid of RRM1 (∆RRM1), RRM2(∆RRM2), or C-terminus (∆C), and the bound HA-VHL was analyzed by Western blotting using anti-HA and anti-FLAG antibodies (**b**) and quantified by the densitometry (**c**). Differences were evaluated by one-way ANOVA (mean ± SD from triplicates; *or #p < 0.05 versus WT). (**d–h**) Identification of E246 in RRM2 as a crucial sequence for recognition by VHL. (**d**) Scheme showing the subdomains in nucleus exporting signal (NES) subdomains (residues 239–250 in human TDP-43). (**e,f** ) WT TDP-43-FLAG, ∆RRM1, ∆RRM2, ∆NES, ∆NES1, ∆NES2, or E246Q/D247N mutants (QN), together with HA-VHL were transfected into HEK293A cells. Cell lysates were immunoprecipitated with anti-FLAG and immunoblotted with anti-HA antibody. Differences were evaluated by one-way ANOVA (mean ± SD from triplicates; *or #p < 0.05 versus WT). (**g,h**) The effect of a single substitution mutant of TDP-43 was similarly analyzed. WT, ∆RRM2, E246Q, D247N, and QN mutants were used. E246Q showed lower affinity to VHL, but D247N did not. (**e**) for IP-FLAG and HA blot, (**f** ) IP-HA and FLAG blot.

We next attempted to identify the site in TDP-43 molecules that is recognized by VHL. First, a pull-down study using domain deletion mutants revealed that RRM2, but not RRM1 or the C-terminus, was required for the interaction with VHL (Fig. 3a–c). Interestingly, elimination of either RRM1 or C-terminus significantly augmented the interaction between VHL and TDP-43 compared with the case of FL WT TDP-43. We next focused on the nucleus export signal (NES), especially on Glu(E)246 and Asp(D)247, which are located at the assembly interface of the RRM2 domain formed upon single-stranded DNA interactions^23^. Moreover, we used the monoclonal antibody 3B12A, which also reacts with C/S mutants of TDP-43 (Supplementary Fig. S5), and showed that D247 was exposed in cytosol-mislocalized TDP-43^24^. We first tested constructs in which various combinations of NESs were eliminated (Fig. 3d). Deletion of any combination of NES residues did not impact the binding affinity between TDP-43 and VHL, which may have been due to the conformation deformity of TDP-43 by NES deletion (Fig. 3e,f). Strikingly, however, E246Q and D247N double mutants, which were constructed to minimize structural changes by side-chain substitution (Fig. 3d–f)^24^, abolished the interaction as well as RRM2 deletion (QN in Fig. 3e,f). Furthermore, we found that E246 is a crucial residue in the interaction, because its substitution to glutamine markedly abolished the binding affinity (Fig. 3g,h). Taken together, these results indicate that E246 and D247 are epitopes for misfolded forms of TDP-43, and E246 is recognized by VHL. The enhanced interaction of VHL with deletion mutants of TDP-43 for C-terminal or RRM1 may also suggest that these domains directly or indirectly cover or block E246 and D247 to prevent them from being exposed.

### CUL2 promotes ubiquitin-mediated degradation of fragmented forms of TDP-43 at proteasomes

**Figure 4:**
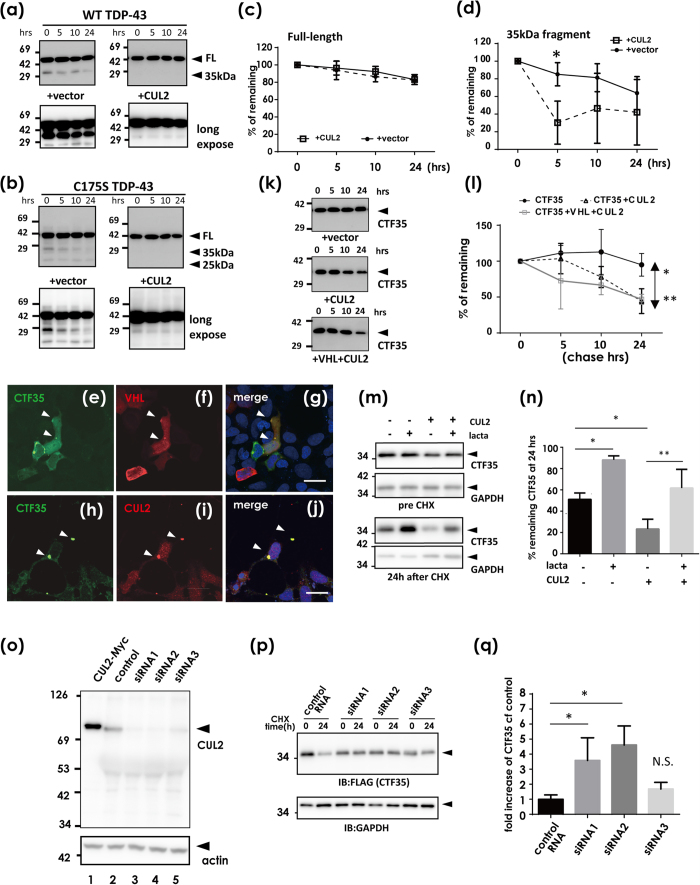
Fragmentation-mediated clearance of TDP-43 by CUL2 E3 ligase. (**a–d**) Protein degradation assay of WT TDP-43 (**a,c,d**) and C175S mutant (**b**) with or without CUL2. At 48 h after transfection of TDP-43-FLAG with or without Myc-CUL2, cells were treated with 100 μg/ml cycloheximide (CHX). The samples were harvested 0, 5, 10, and 24 h after CHX treatment. (**a**) Western blot using anti-FLAG antibodies. (**c,d**) A densitometry for FL (**c**) and 35-kDa (**d**) WT TDP-43. Differences were evaluated by two-way ANOVA (mean ± SD from three independent experiments; *p < 0.05). (**e–n**) CUL2 promotes CTF35 degradation at proteasomes. (**e–j**) Confocal laser micrographs showing the colocalization of CTF35 and VHL (**e,g**) or CUL2 (**h–j**). CTF35-FLAG together with HA-VHL or Myc-CUL2 was transiently co-transfected into HEK293A cells and analyzed by double immunofluorescence. Scale bar, 20 μm. Arrowheads show colocalized inclusions. (**k**) Western blotting for chase study of CTF35 using anti-FLAG antibodies, and densitometry (**l**). Differences were evaluated by two-way ANOVA (mean ± SD from three independent cultures; *p < 0.05, **p < 0.01). (**m,n**) The effect of proteasome inhibition on CUL2-mediated CTF35 clearance. At 48 h after transfection of CTF35-FLAG with or without Myc-CUL2, cells were cultured in the presence or absence of lactacystin (10 μM, 8 h). (**m**) Western blot. (**n**) The average percentage of reduction from the amount of time zero. Differences were evaluated by one-way ANOVA (mean ± SD from three independent cultures; *p < 0.05, **p < 0.01). (**o–q**) Effect of CUL2 knockdown on the amount of CTF35. HEK293A cells were treated with three siRNAs or control oligo for CUL2 for 48 h. (**o**) Western blot using anti-CUL2 antibody. (**p,q**) The chase assay of CTF35 during CUL2 knockdown. (**p**) A representative Western blot using anti-FLAG and anti-GAPDH antibodies. (**q**) The increased ratio of CTF35 in CUL2-knockdown cells compared with control oligo. The similar trends were observed in three independent studies. Differences were evaluated by one-way ANOVA (mean ± SD from triplicate experiments; *p < 0.05).

**Figure 5:**
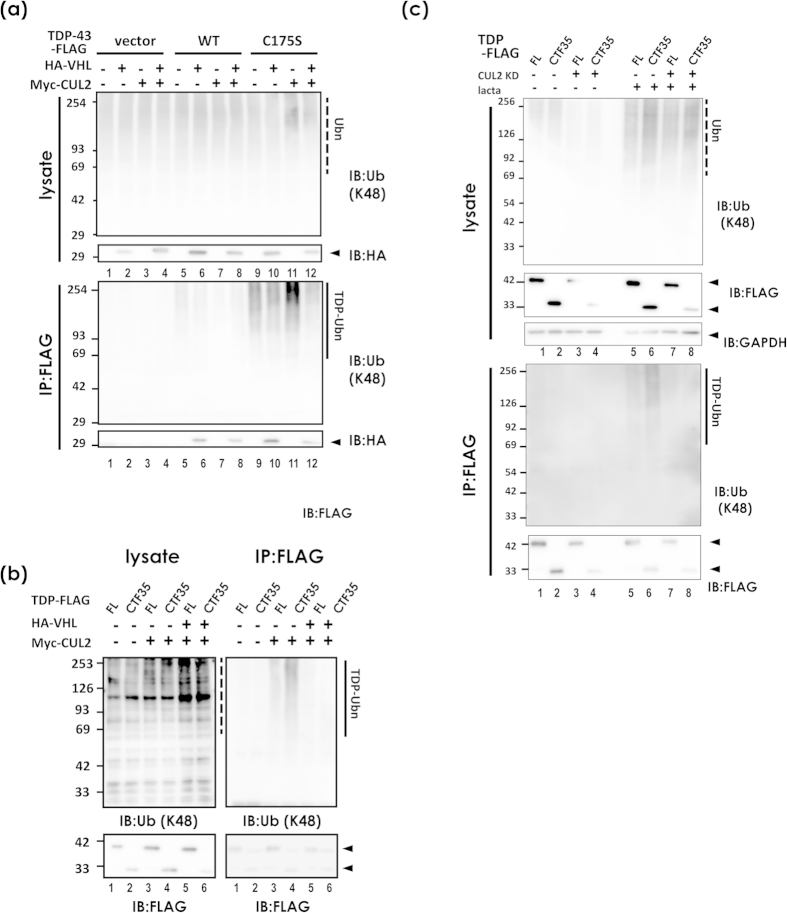
CUL2 ubiquitinates CTF35. (**a**) *In vivo* ubiquitination assay of HEK293A cells co-transfected with HA-VHL, Myc-CUL2, and WT and C175S TDP-43-FLAG. TDP-43-FLAG was pulled down with anti-FLAG affinity beads and blotted with anti-Lys48 ubiquitin. A dashed line indicates total ubiquitin species, while a solid line indicates ubiquitinated TDP-43. Ubn indicates polyubiquitin. (**b**) The same assay showing that CUL2 ubiquitinates CTF35 more than FL TDP-43. Note that double overexpression of VHL and CUL2 reduced the ubiquitinated proteins. A dashed line indicates total ubiquitin species, while a solid line indicates ubiquitinated FL TDP-43 or CTF35. (**c**) CUL2 knockdown inhibited the ubiquitination of CTF35. At 24 h after the introduction of siRNA for CUL2 (siRNA1 in Fig. 4n), FL or CTF35 TDP-43 was additionally transfected. After further incubation for 48 h, lysates were immunoprecipitated with anti-FLAG affinity beads and analyzed by Western blotting for ubiquitin. A dashed line indicates total ubiquitin species, while a solid line indicates ubiquitinated FL TDP-43 or CTF35.

Because the VHL/CUL2 complex is an E3 ligase for HIF1α^15^ or activated atypical protein kinase C^17^, we next investigated whether VHL/CUL2 could degrade TDP-43 through the ubiquitin-proteasome pathway. First, the chase analysis of HEK293A cells transfected with FLAG-tagged TDP-43 after inhibition of protein synthesis by cycloheximide (CHX)^12^ showed no significant effect of CUL2 on the half-life of FL TDP-43 of WT (Fig. 4a,c) or C175S mutant (Fig. 4b). However, we noticed that C-terminal fragments of 35 kDa and 25 kDa were markedly decreased in the presence of CUL2 in the Western blot panels (Fig. 4a,b). Accordingly, the densitometry of the chase study for displayed no significant effect of CUL2 to degrade FL WT TDP-43. However, its 35-kDa fragments showed a trend toward accelerated clearance in the presence of CUL2, which was significant at 5 h after CHX treatment in WT TDP-43 (Fig. 4d). To investigate whether CUL2 promotes the degradation of small fragments of TDP-43 or prevents fragmentation of full-length (FL) TDP-43, we made a construct of the C-terminal 35 kDa form (CTF35) that begins at the previously reported cleavage site^25^. We first confirmed with confocal laser microscope analysis that CTF35 lacking an NLS occasionally formed cytosolic aggregates, which overlapped with co-transfected VHL or CUL2 (Fig. 4e–j). The CHX-chase study revealed that CUL2 significantly promoted the degradation of CTF35 (Fig. 4k,l). The clearance of CTF35 was proteasome mediated, because lactacystin increased the amount of CTF35 (Fig. 4m,n). This effect was reversed by knockdown of CUL2 by siRNA (Fig. 4p,q), and the reversal effect was correlated with the knockdown efficiency of the siRNAs (Fig. 4o,q). An *in vivo* ubiquitination assay showed no clear trend toward increased ubiquitination of FL TDP-43 by CUL2 (Fig. 5a, lanes 10 and 11 in a lower panel). On the other hand, the polyubiquitinated species of CTF35 was more clearly increased by CUL2 (Fig. 5b, lane 3 in the right upper panel). In agreement with this, knockdown of CUL2 by siRNA suppressed the ubiquitination of CTF35 (Fig. 5c, lanes 7 and 8 in the lower panel). We also consistently noticed that co-expression of VHL and CUL2 resulted in decreased ubiquitination of FL or CTF35 of TDP-43 (Fig. 5a,b). Due to the lack of an effect of bafilomycin, an autophagy inhibitor, to prevent autophagy-mediated degradation of TDP-43 (data not shown), we assume that toxicity resulted from overwhelming amounts of ubiquitinated species comprising VHL/CUL2 and TDP-43.

**Figure 6:**
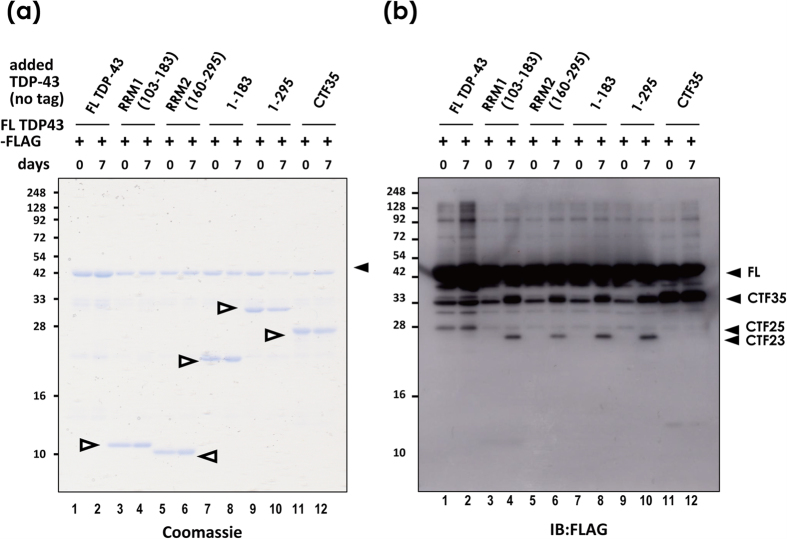
TDP-43 is mechanically fragile and readily fragmented *in vitro*. (**a,b**) Recombinant FL TDP-43-FLAG proteins were incubated with equimolar amounts of various fragments, including RRM1 (aa103–183), RRM2 (160–295), shorter NTF (1–183), longer NTF (1–295), and CTF35 as indicated by open arrowheads. Proteins were denatured immediately after mixing, or after a 7-day incubation at 4 °C. (**a**) Coomassie staining. (**b**) a Western blot using anti-FLAG antibody.

In addition to these active pathways for fragmentation, we also found that recombinant proteins purified from bacteria are mechanically fragile for non-enzymatic cleavage of both CTFs and N-terminal fragments *in vitro*. Notably, 7-day incubation at 4 °C together with CTFs, NTFs, RRM1, or RRM2 domains enhanced the production of CTFs from FL-TDP-43 (Fig. 6, Supplementary Fig. S6).

### Excessive VHL stabilizes and augments the inclusion formation of TDP-43 at JUNQ

**Figure 7:**
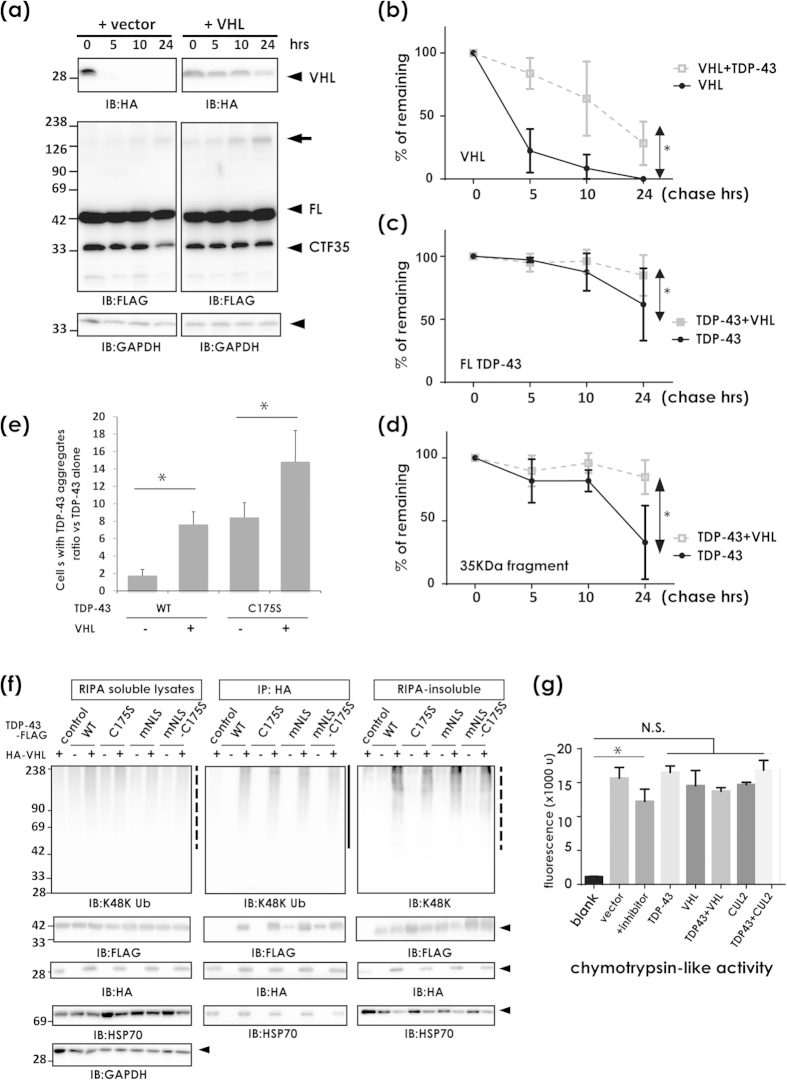
Adverse effect of excess amount of VHL on TDP-43 clearance. (**a–d**) The chase assay of TDP-43 and VHL. (**a**) Western blotting panels using anti-FLAG or anti-HA antibodies. Arrow indicates high molecular weight species of TDP-43 that emerged in the presence of VHL. (**b–d**) Densitometry for VHL (**b**), FL TDP-43 (**c**), and CTF35 (**d**). TDP-43 and VHL stabilize each other. Data are the average from three independent experiments. Differences were evaluated by two-way ANOVA (mean ± SD; *p < 0.05). (**e**) Effect of VHL on TDP-43 aggregation in the cytosol. Under the confocal microscope, TDP-43-positive aggregates were randomly counted at 48 h after transfection of TDP-43-FLAG (WT or C175S mutant) and/or HA-VHL (see Supplementary Figure S7). Data are the average from three independent experiments. Differences were evaluated by two-way ANOVA (mean ± SD; *p < 0.05). (**f** ) Effect of TDP-43 on VHL ubiquitination and aggregation. Total lysates in RIPA buffer were immunoprecipitated with anti-HA affinity beads. RIPA-insoluble fractions were resolved in 8 M urea. The binding complexes with HA-VHL were analyzed by Western blotting with antibodies against ubiquitin Lys48, HSP70, HA, FLAG, and GAPDH. TDP-43-FLAG was more clearly seen in the RIPA-insoluble fraction in the presence of VHL. (**g**) Effect of over expression of TDP-43 and VHL on the proteasomal activity. At 48 h after transfection of expression vectors, cells were harvested and analyzed. Cells transfected with TDP-43, VHL, and CUL2 did not repress proteasome activity. N.S. indicates not significant by one-way ANOVA (mean ± SD from triplicates. *p < 0.05).

In contrast to our evidence that CUL2 ubiquitinated and degraded CTF35, we also noticed that VHL overexpression without CUL2 promoted inclusion formation of TDP-43. Very interestingly, the CHX-chase assay clearly documented that TDP-43 and VHL synergistically prevented the degradation of each other (Fig. 7a–c). Moreover, clearance of the 35-kDa fragments was more affected in the presence of VHL (Fig. 7d). In agreement with this, VHL overexpression significantly increased the number of cells containing perinuclear inclusions, more prominently for aggregate-prone TDP-43 (Fig. 7e, Supplementary Fig. S7). Furthermore, TDP-43 promoted the ubiquitination of VHL regardless of the type of TDP-43 (Fig. 7f). TDP-43 also promoted the accumulation of VHL in insoluble fractions (Fig. 7f, the right panels). VHL interacted with Hsp70 in reagent for immunoprecipitation assay (RIPA)-soluble fractions and in the inclusions (Fig. 7f, the left panel Supplementary Fig. S8), indicating that these perinuclear inclusions have the molecular features of JUNQ, a temporary storage compartment for ubiquitinated proteins before processing in the 26S proteasome^26^,^27^. Hsp70 accumulated less in the urea-soluble fractions when TDP-43 and VHL were co-transfected (Fig. 7f, the right bottom). Proteasome activity measured as chymotrypsin activity was not impaired by overexpression of both WT TDP-43 and VHL (Fig. 7g).

### TDP-43 negatively regulates VHL expression

**Figure 8:**
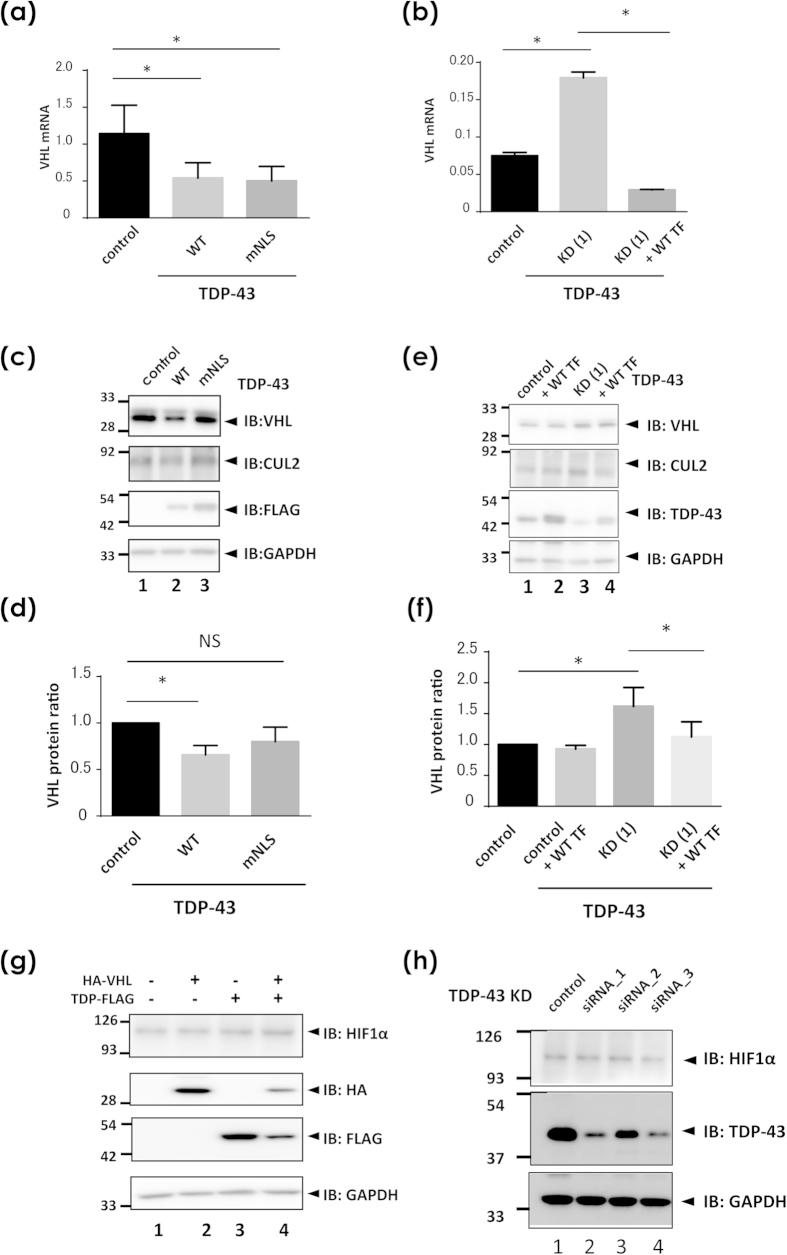
The role of TDP-43 in the expression of VHL, CUL2, and HIF1α. (**a–f** ) Measurement of VHL in the presence of overexpression (**a,c,d**) or knock down (**b,e,f**) of TDP-43 in HEK293A cells. (**a,b**) Quantitative real-time PCR analysis of VHL expression in the presence of overexpression or knock down of TDP-43. HEK293A cells were transiently transfected with WT or mutant NLS TDP-43 (**a**) or siRNA oligonucleotides targeting TDP-43 (**b**). At 48 h and 96 h after transfection in (**a**) and (**b**), respectively, cells were harvested for cDNA generation from RNA. In (**b**), plasmid for WT TDP-43 was additionally introduced at 48 h before harvest for the rescue experiment. Each data point represents the average from three (**a**) and six (**b**) cultures (mean ± SD; *p < 0.05). N.S. indicates not significant by one-way ANOVA. (**c–f** ) Western blotting for protein levels of endogenous VHL and CUL2 in the presence of overexpression (**c,d**) or knock down (**e,f** ) of TDP-43. After the same experiments as in (**a,b**), cell lysates were analyzed by Western blotting using antibodies against CUL2, VHL, TDP-43, and GAPDH. At 48 h and 96 h after transfection in (**a,b**), respectively. As same as in (**b**), WT TDP-43 was additionally transfected at 48h before harvest for the rescue experiment. Western blotting panels for endogenous VHL, CUL2, GAPDH (**c,e**), and TDP-43-FLAG (**c**) or endogenous TDP-43 (**e**). (**d,f** ) are densitometric data for (**c,e**), respectively. Each data point represents the average from four cultures of cells (mean ± SD; *p < 0.05). N.S. indicates not significant by one-way ANOVA. (**g,h**) Western blotting showing the effect of TDP-43 overexpression (**g**) or knock down (**h**) on HIF1α expression in HEK293A cells. No significant effect was observed in HIF1α levels in either condition of TDP-43.

We next investigated the effect of TDP-43 on the expression of VHL in HEK293A cells. First, we measured VHL mRNA with real-time PCR analysis and protein levels with Western blotting and demonstrated negative regulation of VHL by TDP-43. Overexpression of WT TDP-43 induced a reduction in both mRNA and protein levels of VHL (Fig. 8a,c,d). TDP-43 carrying mutant NLS (mNLS) suppressed VHL transcription, but this was not observed at the protein level. On the other hand, RNA knockdown of TDP-43 resulted in upregulation of both transcription and protein levels of VHL as observed with real-time PCR (Fig. 8b) and Western blot analysis (Fig. 8e,f), respectively. Off-target effects were excluded, because rescuing cells with additional TDP-43 overexpression in TDP-knockdown cells reversed the level of VHL transcription (Fig. 8b). The reverse effect of the rescue plasmid in the Western blots is mild (Fig. 8e); it is possible proteasome might be impaired by 48 h knockdown of TDP-43 before rescue transfection, as well as insufficient time for the reversal. These results indicate that the negative regulation of VHL by TDP-43 may underlie the inclusion pathology associated with nuclear exclusion of TDP-43. Western blotting for HIF1α showed no obvious effect of TDP-43 expression or knockdown on the HIF1α levels (Fig. 8g,h).

### Phosphorylated TDP-43 and VHL are colocalized in cytoplasmic inclusions in oligodendrocytes in ALS

**Figure 9:**
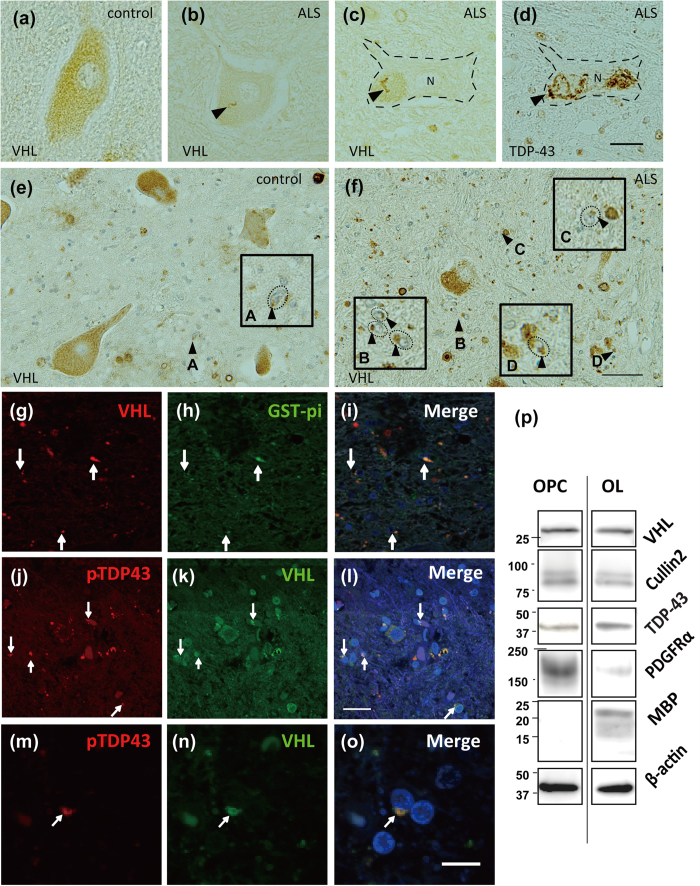
Immunohistochemistry showing the colocalization of phosphorylated TDP-43 and VHL in the oligodendrocytes in ALS spinal cords. Immunohistochemistry of spinal motor neurons (**a–d**) and oligodendrocytes (**e–n**) for VHL (**a–c,e,f** ) and TDP-43 (**d**) of the spinal cords from ALS patients (**b–d**,**f–n**) and ALS-irrelevant control subjects (**a,e**). Sections (**c,d**) are serial slices from one block. In a subset of motor neurons of ALS patients, string-like appearance of VHL (**b,c**) occasionally colocalized with TDP-43 skeins in part (arrows in c and d). In (**c,d**), a motor neuron was demarcated by dotted line for the clarity. Scale bar = 20 μm. In (**f** ), cytoplasmic aggregates of VHL in oligodendrocytes are indicated by arrowheads, which were magnified with demarcation by dotted line in the insets (**A–D**). (**g–o**) Double immunofluorescence study shows that the VHL colocalize with Glutathione-S-transferase (GTS) pi, a marker for oligodendrocytes (**g–i**), and with phosphorylated TDP-43 (**j–l**, **m–o**) in cytoplasmic inclusions (arrows). Scale bar = 50 μm. (**p**) Western blotting of the cell lysates from cultured precursor oligodendrocytes (OPC) and mature oligodendrocyte (OL) for VHL, CUL2, and TDP-43. As a marker for OPC and OL, antibodies against PDGFRα and myelin basic protein (MBP) were used, respectively.

By immunohistochemistry, we demonstrated that VHL is expressed in the cytosol of spinal motor neurons in both ALS and control subjects (Fig. 9a). In several motor neurons in ALS, string-like structures, especially in lipofuscin granules were VHL-positive (Fig. 9b–d), and occasionally colocalized with TDP-43 (Fig. 9c,d) in part. However, VHL expression in motor neurons was not so abundant as expected. On the other hand, we noticed VHL frequently formed inclusions in oligodendrocytes, namely glial cytoplasmic inclusions (GCIs) more in the ALS patients (Fig. 9e,f, Supplementary Figure S9b, see highly magnified GCIs in insets). Double immunofluorescence study revealed that VHL frequently colocalized with glutathione-S-transferase (GST) pi, a marker of oligodendrocytes (Fig. 9g–i, Supplementary Fig. S9), and with phosphorylated TDP-43(Fig. 9j–l,m–o, Supplementary Fig. S9c-f, g-j), verifying their presence predominantly in oligodendrocytes in the ALS spinal cords. We also confirmed VHL and CUL2 were expressed in oligodendrocytes of both precursor cells and mature types, using the cell lysates from primary cultures from embryonic rat brains (Fig. 9p).

### VHL interacts with and increases inclusion formation of ALS-linked mutant SOD1

**Figure 10:**
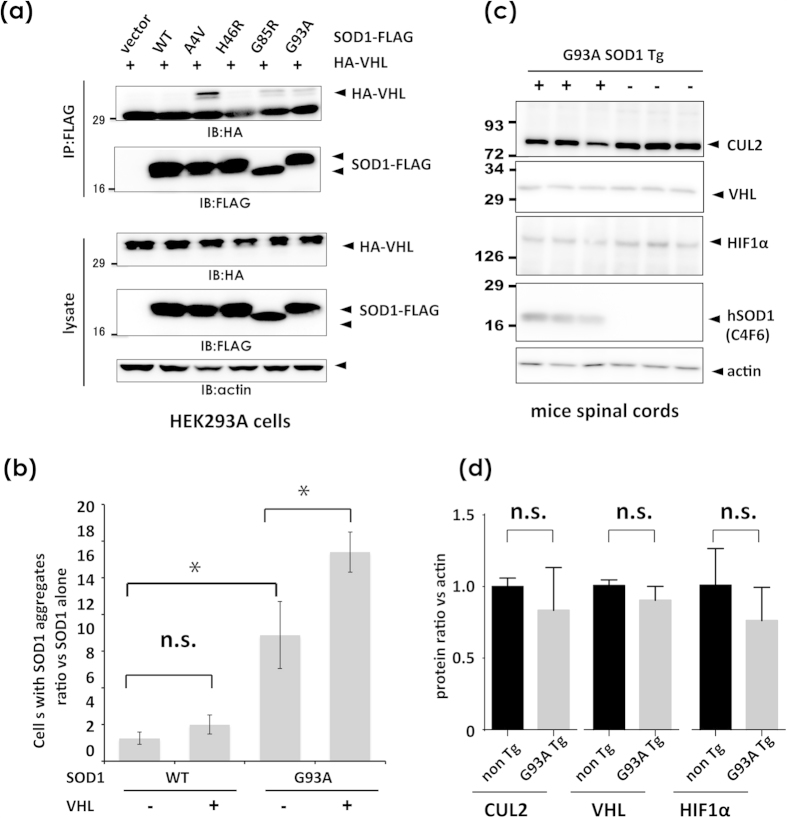
VHL interacts with ALS-linked SOD1 mutants, and promotes the aggregation. (**a**) Interaction of VHL with ALS-linked mutant SOD1 proteins. Lysates from HEK293A cells transfected with HA-VHL and WT or ALS-linked mutant SOD1-FLAG (A4V, H46R, G85R, and G93A) were immunoprecipitated with anti-FLAG affinity beads and analyzed by Western blotting using anti-HA antibody. (**b**) At 48 h after transfection of SOD1-FLAG (WT or G93A mutant) and/or HA-VHL, cells were fixed and stained as shown in Fig. 1. The number of cells containing SOD1-positive aggregates were counted (see supplementary Figure S10). VHL promoted the formation of SOD1 aggregates. Data are the average from three independent experiments. Differences were evaluated by one-way ANOVA (mean ± SD; *p < 0.05). n.s. indicates not significant (*p* = 0.0533). (**c,d**) Western blot analysis of VHL, CUL2 and HIF1α in the spinal cord lysates from mutant SOD1 transgenic mice. The tissue lysates from spinal cords of Tg mice of 280 days old, and non-transgenic mice of 230 days old (three mice each), were analyzed by blot images (**c**) and densitometry 8 d). The difference between the non Tg and G93A Tg in each protein was assessed by Stuent’s t test. n.s. indicates not significant.

Previous work by Weisberg *et al.* documented that VHL overexpression induces JUNQ immobilization of mutant SOD1, which may underlie the pathogenesis of mutant SOD1-linked ALS^28^. Based on the effect of excess VHL on TDP-43 accumulation, the interaction between VHL and mutant SOD1 was investigated using transfected cell lysates. Western blotting analysis revealed co-immunoprecipitation of mutant SOD1 with VHL, but not WT or long-lived mutant SOD1 H46R (Fig. 10a). Moreover, VHL significantly increased the number of inclusion-harboring cells only with mutant SOD1 (Fig. 10b, Supplementary Fig. S10). We further analyzed the expression pattern of VHL, CUL2 and HIF1α in the spinal cord lysates from mutant SOD1 transgenic mice at early symptomatic age. These three molecules were abundantly expressed in the spinal cords in comparative expression levels between transgenic and non-transgenic mice. Interestingly, CUL2 and HIF1α levels had a trend of decrease in transgenic mice compared with non-transgenic mice at the near age, although not significant (Fig. 10c,d).

## Discussion

We identified the VHL/CUL2 E3 complex as a novel binding partner with TDP-43. VHL distributes in various regions of the central nervous system, including Purkinje cells, Golgi type II cells, and cells in the dentate nucleus of the cerebellum, pontine nuclei, and inferior olivary nucleus of the medulla oblongata^29^. Spinal anterior horn cells express VHL, as shown by *in situ* hybridization^30^ and immunohistochemistry with monoclonal antibodies^31^. It is also reported that VHL/CUL2 is critically involved in the maturation of oligodendrocytes^32^. In the context of neurodegenerative diseases, VHL interacts with DJ-1 and prevents HIF1α degradation by CUL2, which may partly explain the neuroprotective role of DJ-1 in Parkinson’s disease^33^.

**Figure 11:**
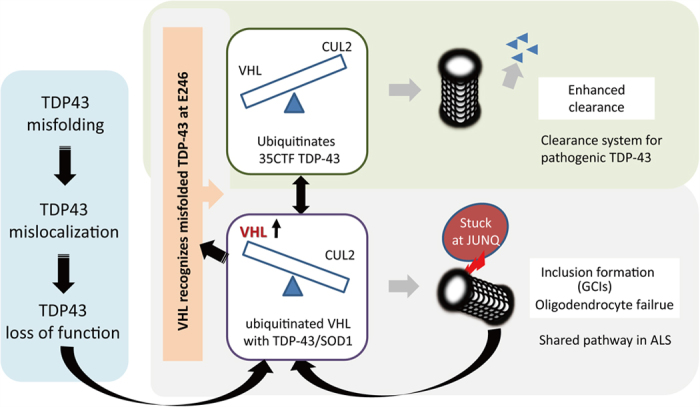
A hypothetical scheme regarding the role of VHL in TDP-43 proteinopathy. In cases of TDP-43 misfolding or unfolding such as in stress conditions, E246 would be exposed and recognized by VHL. VHL together with CUL2 would preferentially degrade fragmented forms of TDP-43 through ubiquitination. Conversely, excess amounts of VHL promote a formation of TDP-43 or mutant SOD1 inclusions at JUNQ. The loss of TDP-43 function by the misfolding or mislocalization, induce the expression of VHL.

Interestingly, VHL preferentially recognized misfolded forms of TDP-43. The enhanced interaction between VHL and TDP-43 under the paraquat challenge, which induces stress granules^22^, implies the involvement of VHL in the TDP-43 proteinopathy in ALS. We further identified E246 in TDP-43 as a crucial residue for the interaction with VHL. This sequence is present in the NES in RRM2 and works together with D247 as a self-assembly interface of RRM2 domains upon DNA interaction^23^. The exposure of D247 in the misfolded TDP-43 was demonstrated by our monoclonal antibody 3B12A, which recognizes mislocalized TDP-43 in cultured cells, the skein-like or round inclusions in ALS patients^24^, and aggregated forms of TDP-43 with C173/C175 substitutions (Supplementary Fig. S5). These results suggest that the residues E246 and D247 are molecular epitopes for misfolded TDP-43, and that TDP-43 may be recognized by VHL once it is unfolded or misfolded in the cytosol (Fig. 11).

VHL recognizes hydroxylated proline residues of HIF1α under normoxic conditions^34^. However, our results together with previous reports describing the interaction of VHL with active protein kinase C^17^ or with DJ-1 regardless of the proline residues, strongly indicate the presence of other recognition machineries for VHL. The chase study for overexpressed WT TDP-43 showed unexpected results, in which fragmented forms such as 35- or 25-kDa fragments were more efficiently cleared by CUL2 than FL WT TDP-43. Moreover, the degradation assay using CTF35 in the presence of overexpressed or knocked down VHL confirmed that fragmented forms of TDP-43 are substrates of the VHL/CUL2 complex. Although the reason that full-length TDP-43 was resistant to CUL2 is unknown, the N-terminus may play a role in preventing VHL access or E3 ligase activity of the CUL2 complex. Earlier research documented the presence of CTFs of 35 or 25 kDa in patients with TDP-43 proteinopathy, and numerous subsequent works have demonstrated their pathogenic roles both *in vitro* and *in vivo*. The machinery that produces these CTFs includes caspases^35^,^36^, other proteases^37^, or even alternative splicing^38^. We also found that recombinant FL TDP-43 is unstable when isolated and is readily fragmented non-enzymatically, together with small fragments (Fig. 6, Supplementary Fig. S6). These *in vitro* data imply that TDP-43 is in a high-energy state in its FL form, whereas fragmented forms are more stable structurally. Taken together, fragmentation may play an important role in the degradation of misfolded forms of FL TDP-43. In this work, we have focused on CTF35 as a target species of CUL2, because the amount of CTF25 in our culture paradigm was too small to be quantitatively assessed. However, our chase studies for FL TDP-43 (Fig. 4) showed a clear trend toward elimination of CTF25s by CUL2 overexpression. We thus assume that CUL2 is involved in fragment-specific clearance of TDP-43. Considering the pathological evidence that the majority of TDP-43 inclusions lack the N-terminus^39^, we are tempted to assume that self-assembly in the inclusions would yield the fragments of TDP-43.

On the other hand, the overexpression of VHL unexpectedly stabilized TDP-43, especially the fragmented species, and accelerated aggregate formation of TDP-43 (Fig. 7a–e). Proteasome activity was shown to be preserved by the chymotrypsin activity assay (Fig. 7g). In contrast to its proteolytic role, VHL is aggregate-prone and is localized in perinuclear inclusions called JUNQ^26^. In both yeast and mammalian cells, misfolded proteins accumulate into stress foci and are partitioned into “insoluble protein deposit” (IPOD) and JUNQ, depending upon their state of ubiquitination^28^,^40^. IPOD contains non-ubiquitinated aggregate-prone proteins in non-stressed cells^40^, whereas JUNQ is a temporary storage cargo before degradation of the ubiquitinated proteins in adjacent 26S proteasomes. Heat shock chaperones such as Hsp107 and Hsp70 are recruited to JUNQ to disaggregate misfolded proteins for processing through the ubiquitin-proteasome system. Tat-binding protein-1, a component of the 19S proteasome component, interacts with VHL and promotes the ubiquitin-dependent proteasome degradation of HIF1α^41^. This may imply that VHL is involved in the formation of JUNQ in mammalian cells^42^. Our data indicate synergistic effects of VHL and TDP-43 in the formation of inclusions at JUNQ, which may underlie TDP-43 cytopathologies in ALS. It is also conceivable that aberrant interaction between excessive VHL and misfolded TDP-43 possibly stabilizes with each other, and may lead to prevent them from releasing from JUNQ, as is reported in the case of mutant SOD1^28^. Moreover, knockdown of TDP-43 promoted the transcription and translation of VHL (Fig. 8). This is in agreement with the notion that mislocalized TDP-43 causes a knockdown effect for TDP-43^43^, which may allow the interaction between elevated VHL and mislocalized TDP-43 in the cytosol. Although the overexpression of mNLS TDP-43, suppressed the VHL transcription as well as WT TDP-43, this could happen since our mNLS constructs keep Lys97/Arg98, and partially distributes in the nucleus^12^. The inconsistency of the VHL levels between mRNA and proteins, might be ascribable to several factors, including proteasome condition under long-term overexpression or knockdown of TDP-43, or antibody affinity. Interestingly, VHL interacted with and promoted inclusion formation of mutant SOD1, but not WT or long-lived mutant H46R SOD1, implying a common role of VHL in ALS proteinopathy (Fig. 10). The VHL/CUL2 complex is an E3 ligase for HIF1α, and an immunohistochemical study of spinal cord slices from ALS patients showing mislocalized HIF1α in the cytosol^44^ might be ascribable to the VHL/CUL2 system. However, despite that TDP-43 is a negative regulator of VHL, neither overexpression nor downregulation of WT TDP-43 impacted the amount of HIF1α (Fig. 8g,h). This apparent discrepancy might imply the presence of degradation machineries other than the VHL/CUL2 complex, such as glycogen synthase kinase 3^45^.

Here we demonstrated that VHL is crucially involved in the formation of cytoplasmic inclusions in oligodendrocytes (namely glial cytoplasmic inclusions; GCIs) rather than in motor neurons by immunohistochemistry. Why TDP-43 inclusions in motor neurons and oligodendrocytes showed different colocalization patterns for VHL remains elusive, the abundant expression and the more active roles of VHL/CUL2 in oligodendrocytes for their maturation from OPCs could underlie the interaction between VHL and misfolded TDP-43 than in motor neurons^32^. Oligodendrocytes are among target cells for inclusions of ALS-linked proteins such as phosphorylated TDP-43 and FUS, and recently has attracted much attention since they play important roles in ALS pathogenesis, for instance, by the dysfunction of monocarboxylate transporter 1 (MCT1)^18^,^19^,^20^. Although the underlying pathomechanisms remain elusive, the imbalance of VHL/CUL2 might affect oligodendrocytes in ALS related to the accumulation of pathogenic proteins, including TDP-43 and mutant SOD1.

## Conclusions

In conclusion, we identified VHL/CUL2 proteins as a novel E3 ligase for misfolded forms of TDP-43. The substrate-binding protein, VHL, plays bidirectional roles by promoting ubiquitin-mediated degradation of fragmented TDP-43 at proteasomes with CUL2, and by stabilizing TDP-43 at JUNQ when upregulated. The deregulation of this pathway would cause oligodendrocyte failure in ALS. Further investigation of regulation of the VHL/CUL2 balance may suggest therapeutic strategies for ALS.

## Methods

### Reversible covalent-linked immunoprecipitation coupled with *in vitro* ubiquitination

TDP-43-interacting proteins during the ubiquitin-proteasome reaction (UPR) were investigated with a combination of the reversible covalent-linked immunoprecipitation (ReCLIP) assay^21^ and *in vitro* ubiquitination^46^ with several modifications, in which recombinant human TDP-43-FLAG proteins were incubated with ubiquitination reaction solution containing detergent-soluble S100 lysates from HEK293A cells. The ubiquitination mixture comprising recombinant TDP-43, S100 lysates, purified ubiquitin, and ATP generating system (Substrate 0.1–1 μg, 20 mM Tris pH 7.5, 5 mM MgCl_2_, 2 mM DTT, 0.5 μg ubiquitin aldehyde (Boston Biochem, Cambridge, MA), 5 μg ubiquitin, 30 μg S100 lysate 0.5 mM ATP, 10 mM creatine phosphate, and 0.5 μg of creatine phosphokinase in a total volume of 20 μl) was incubated for 30 min at 37 °C. The disulfide-dependent cross-linker, Dithiobis[succinimidyl propionate] (DSP), was then added to the mixture at a concentration of 40 mM and was allowed to cross-link for another 30 min. Disulfide formation was terminated with excess l-cysteine (20 mM final). TDP-43-FLAG proteins were pulled down with anti-FLAG affinity beads (M2, Sigma St. Louis, MO), and disulfide-mediated binding proteins were released by mild reducing conditions (50 mM DTT at 37 °C for 30 min). The eluates were separated on an acrylamide gel and visualized with a fluorescent dye (Oriole, BIO-RAD, Hercules, CA). Positive bands, which disappeared in the absence of ATP or TDP-43-FLAG, were analyzed by liquid chromatography-tandem mass spectrometry (LC-MS/MS), as previously described^47^.

### Antibodies

Rabbit polyclonal anti-DYKDDDD antibody and mouse anti-GFP antibody were purchased from Cell Signaling Technology (Beverly, MA) and Nacalai Tesque (Kyoto, Japan), respectively. Rabbit polyclonal anti-Cullin2 (CUL2) and anti-TDP-43 antibodies were purchased from Abcam (Cambridge, UK) and Proteintech (Chicago, IL), respectively. Rat monoclonal anti-HA antibody was obtained from Roche (Basel, Switzerland). Mouse monoclonal antibodies against actin (C4), elongin B (D-5), and elongin C (56) were obtained from Santa Cruz Biotechnology (Santa Cruz, CA). Mouse monoclonal anti-FLAG antibody (M2) was obtained from Sigma. Rabbit anti-ubiquitin, Lys48-specific antibody was obtained from EMD Millipore (Billerica, MA). Mouse anti-Hsc70/Hsp70 antibody was purchased from StressGen (Victoria, BC). Mouse anti-HIF1α antibody was obtained from Abcam. Goat anti-mouse IgG Alexa fluor488 and fluor405 were purchased from Invitrogen (Carlsbad, CA), and goat anti-rabbit IgG CF568 was from BIOTIM (Hayward, CA). Mouse monoclonal anti-human VHL was generated as previously described^31^.

### Plasmid construction

Mammalian expression plasmids for TDP-43 tagged with FLAG (pcDNA3-TDP-43-FLAG) or EGFP (pEGFP-N3-TDP-43) were constructed using a conventional PCR technique as described previously^12^. TDP-43 with substitution mutants for cysteine with serine at Cys173 and/or Cys175 (C175S, C173S/C175S; DCS), and familial ALS-linked mutations (A315T and Q331K) and mNLS were generated using site-specific mutagenesis^12^. In several experiments, target proteins were driven under the CAGs promoter^34^. Plasmids for TDP-43 with mutations in RRM2 were made with site-specific mutagenesis^24^. Deletion mutants of TDP-43 for RRM1 (ΔRRM1), RRM2 (ΔRRM2), NES (ΔNES), or the C-terminal region (ΔC) were generated by PCR with primer pairs used to delete the nucleotides encoding these domains^11^. Plasmids of TDP-43 for NES1 (ΔNES1, deletion of aa 239–243) and NES2 (ΔNES2, deletion of aa 246–250) were also made with site-specific mutagenesis (Agilent Technologies, Santa Clara, CA).

The mutant C-terminal fragment 35 kDa (CTF35, aa 90–414) tagged with FLAG (pcDNA3-CTF35-FLAG) and CUL2 tagged with Myc (pCMV-Myc-CUL2) were constructed using conventional PCR and subcloned into pcDNA3-FLAG using the BamHI/XhoI sites or pCMV-Myc using the BamHI/KpnI sites. Human VHL tagged with HA (pHM6-HA-VHL) was constructed using conventional PCR and subcloned into pHM6-HA using the HindIII/EcoRI sites. The primer pairs are described in Supplementary Table 2.

### Cell culture, transfection, RNA knockdown

All cultured cells were maintained at 37 °C in 5% CO_2_ and 100% humidity. HeLa cells and HEK293A cells (Invitrogen) were maintained in Dulbecco’s modified Eagle’s medium (Nacalai) containing 10% fetal bovine serum and penicillin/streptomycin (Nacalai). Lipofectamine^TM^ 2000 (Invitrogen) and FuGene HD Transfection Reagent (Promega Fishberg, WI) were used for transfection of the plasmid according to the manufacturer’s protocols. For the RNA knockdown experiment, short interference RNA pairs were transduced into cells using Lipofectamine2000 and Magnetofection system (CombiMag, OZ Bioscience, San Diego, CA). Incubation time for the overexpression and siRNA knockdown transfection before the harvest, cells were 48 h and 96 h, respectively, unless mentioned.

### Western blotting, immunoprecipitation, and *in vivo* ubiquitination

At 48 h after the transfection, cultured cells were lysed in co-immunoprecipitation buffer (co-IP, 20 mM HEPES pH 7.4, 125 mM NaCl, 2 mM EDTA, 1% Triton X-100, 10% Glycerol) or RIPA buffer (20 mM HEPES-KOH pH 7.4, 150 mM NaCl, 2 mM EDTA, 1% Nonidet-P40, 1% sodium deoxycholate), containing protease inhibitor cocktail (Roche) and PR-619 (LifeSensors, Malvern, PA), a de-ubiquitination inhibitor for ubiquitination assay. In several experiments, RIPA-insoluble fractions were solubilized in 8 M urea. Ten percent of each cell lysate was analyzed as the total cell lysate, and the remaining 90% was incubated with anti-FLAG M2 affinity gel (Sigma) or beads coated with anti-HA antibody (Wako, Kyoto, Japan) at 4 °C overnight. The immunoprecipitates were eluted into sodium dodecyl sulfate (SDS) sample buffer. Samples were reacted in one-third volume of 4× SDS buffer, denatured at 95 °C for 5 min, separated on a 5–20% or 15% polyacrylamide gel (Wako), and transferred onto a PVDF membrane (Millipore) for Western blotting. Proteins were detected using an enhanced chemiluminescence system (ECL, Thermo-Fisher Scientific, Waltham, MA or Nacalai), and the densitometric analysis of protein was performed using Image J software^48^.

### Immunofluorescence and microscopic analysis

At 48 h after the transfection, cultured cells were fixed in 4% paraformaldehyde in PBS, pH 7.2, for 20 min at room temperature and permeabilized with 0.1% Triton X-100 containing 5% normal goat serum as blocking agent. Cells were reacted with primary antibody (4 °C overnight) and subsequently with fluorescence-tagged secondary antibody (Alexa, Invitrogen, or CF, Sigma) for 1 h at room temperature. Cells were counter-stained with 4′,6-diamidino-2-phenylindole (DAPI). Fluorescent images were obtained using a confocal laser microscope FV1000-D IX81 (Olympus, Tokyo, Japan) or fluorescence microscope (BZ-X700, Keyence, Osaka, Japan).

Information about the antibodies used is described in Supplementary Table 3.

### Degradation assay

Protein half-life was estimated 48 h after transfection by chronological chase analysis at 5, 10, and 24 h after CHX treatment (100 μg) of HEK293A cells to inhibit protein synthesis. The cells were directly lysed in the SDS sampling buffer containing 100 mM DTT and were denatured at 70 °C for 20 min. The remaining amount of protein as estimated by densitometry was expressed as the percentage of the residual protein level compared with that at the starting point (0 h). To determine the effect of CUL2 on transgene expression, cells were transfected with siRNAs for CUL2 (Stealth RNA I, Invitrogen), incubated for a further 48 h, and subcultured before transfection with TDP-43 plasmids for the chase study.

### Quantitative real-time PCR

The total RNA samples were purified from the transfected cells using a commercially available kit (Invitrogen) and were converted to cDNA with reverse transcriptase (Toyobo, Tokyo, Japan). The levels of mRNAs for VHL and glyceraldehyde 3-phosphate dehydrogenase (GAPDH) in the synthesized cDNAs were analyzed with the SYBR quantitative PCR kit (Toyobo) and real-time PCR Detection Systems (BIO-RAD). GAPDH was used as an internal standard, and the relative expression levels of mRNAs were calculated by the ΔCT method according to the manufacturer’s protocol using the included software (BIO-RAD). The primer sequences are described in Supplementary Table 2.

### Measurement of proteasome activity

Chymotrypsin-like activity was measured as proteasome activity using a commercially available kit (Cayman Chemical Company, Ann Arbor, MI). HEK293A cells were seeded in 6-cm culture dishes at a density of 1 × 10^6^ cells/dish. At 48 h after the transfection, all lysates were analyzed for proteasome activity, which was determined at 360 nm excitation and 480 nm emission using a multi-plate reader (Perkin Elmer, Waltham, MA).

### Immunohistochemistry and immunoblotting of human tissues

All experiments were approved by and performed under the guidelines of the Kyoto University ethics committee. Informed consent was obtained from all individuals or their guardians before the autopsy analysis. Postmortem lumbar spinal cords from five patients with a definite diagnosis of sporadic ALS and five neurological disease controls other than ALS (myotonic dystrophy, cerebral infarction, Parkinson’s disease, and chronic lymphocytic leukemia) were analyzed. At autopsy, spinal cord blocks from the lumbar level were paraffinized after fixation in 10% buffered formalin. Sections with 6 μm-thickness were deparaffinized and antigen retrieved by autoclaving (20 min at 120 °C), using Histofine deparaffinizing antigen retrieval buffer, pH 6 (product code 415281, Nichirei, Tokyo, Japan). Sections were then incubated overnight with primary antibodies in PBS containing 3% bovine serum albumin at 4 °C. The bound antibodies were visualized by the peroxidase polymer-based method using a Histofine Simple Stain MAX-PO kit (Nichirei) with diaminobenzidine as the chromogen. Double immunofluorescence was performed with the similar protocols, except fluorescence-tagged secondary antibodies were used, as mentioned above.

### Primary culture of oligodendrocytes and their precursor cells (OPCs)

Primary cultures of embryonic rat oligodendrocytes and their precursor cells (OPCs) were prepared as previously descried^49^. In brief, cerebral cortices from 1–2 day old Sprague Dawley rats were dissected, minced, and digested. OPCs were purified by the floating culture, in which cells were cultured for 10 days on poly-D-lysine-coated 75 cm2 flask in DMEM containing 20% FBS and 1% penicillin/streptomycin (P/S) until reaching 100% confluence, shaken for 1 h on an orbital shaker (218 rpm) at 37 °C to remove microglia, and were further shaken for additional overnight with newly exchanged medium (~20 h). After the medium was collected and plated on non-coated tissue culture dishes for 1 h at 37 °C to eliminate contaminating astrocytes and microglia, the non-adherent cells were collected and replated in Neurobasal (NB) medium containing 2 mM glutamine, 1% P/S, 10 ng/ml PDGF, 10 ng/ml FGF, and 2% B27 supplement onto poly-DL-ornithine-coated plates. Four to five days after plating, the OPCs were used for the experiments. Mature oligodendrocytes were obtained by differentiating OPCs by exposure to DMEM containing 1% P/S, 10 ng/ml CNTF, 15 nM T3, and 2% B27 supplement (Invitrogen).

### Animal breeding and tissue sampling

Transgenic mice harboring human G93A SOD1 (B6SJLTgN[SOD1-G93A]1Gur, SOD1G93A; Jackson Laboratory, Bar Harbor, ME) were housed and bred as previously reported (Takeuchi *et al.* 2010). The spinal cord was homogenized in a tissue lysis buffer (20 mM HEPES–KOH (pH 7.4), 120 mM NaCl, 2 mM EDTA, 10% glycerol, 1% Triton X-100, and protease inhibitor cocktail) and then incubated for 1 h at 4 °C, and the supernatant was collected for Western blotting. The protein amount was adjusted according to Bradford assay (Bio-rad, Hercules, CA). All study protocols were performed respecting the dignity of animal lives and were approved by the animal experimental committee of the Shiga University of Medical Science (Document# 2009-12-2).

### Statistical analysis

Multiple comparisons were analyzed with one-way ANOVA of Tukey’s multiple-comparison test, and factor estimation in the two chorological data group was studied with two-way ANOVA using Prism software (GraphPad, La Jolla, CA). The difference between the two groups was assessed by Stuent’s t test. *p* < 0.05 was considered statistically significant.

## Additional Information

**How to cite this article**: Uchida, T. *et al.* CUL2-mediated clearance of misfolded TDP-43 is paradoxically affected by VHL in oligodendrocytes in ALS. *Sci. Rep.* **6**, 19118; doi: 10.1038/srep19118 (2016).

## Supplementary Material

Supplementary Information
