# Supplementary material for: CUL2-mediated clearance of misfolded TDP-43 is paradoxically affected by VHL in oligodendrocytes in ALS
Source: Sci Rep. 2016 Jan 11;6:19118. doi: 10.1038/srep19118 (PMC4707540; doi:10.1038/srep19118)
Supplement: Supplementary Information [file srep19118-s1.pdf]

SUPPLEMENTAL MATERIALS

CUL2-mediated clearance of misfolded TDP-43 is paradoxically affected by VHL in oligodendrocytes in ALS

Tsukasa Uchida, M.D.[1], Yoshitaka Tamaki, M.D.[1], Takashi Ayaki, M.D., Ph.D.[1], Akemi Shodai, M.S.[1], Seiji Kaji, M.D.[1], Toshifumi Morimura, Ph.D.[2], Yoshinori Banno, M.D.[2], Kazuchika Nishitsuji, Ph.D.[3], Naomi Sakashita, M.D., Ph.D.[3], Takakuni Maki, M.D., Ph,D.[1],.Hirofumi Yamashita, M.D., PhD. [1], Hidefumi Ito, M.D., Ph.D.[4], Ryosuke Takahashi, M.D., Ph.D.[1], *Makoto Urushitani, M.D., Ph.D.[1]

1. Department of Neurology, Kyoto University Graduate School of Medicine
2. Molecular Neuroscience Research Center, Shiga University of Medical Science
3. Department of Molecular Pathology, Tokushima University
4. Department of Neurology, Wakayama Medical University

Supplementary Table S1; LC-MS/MS results for the proteins, potentially interacting with TDP-43 during ubiquitination.

Protein	accession	P	Score	coverage	Sf	Functions
hCG1811380, isoform CRA_b	EAW79886	0.00006	10.1	1.2	0.03	Protein Tyrosine Kinases, Class EphA Ephrin Receptors, Ligand Binding Domain of Ephrin type-A Receptor 6
cullin-2 isoform c	19482174	0.00008	10.1	2.6	0.28	cullin-RING ubiquitin ligase complex, G1/S transition of mitotic cell cycle induction of apoptosis by intracellular signals, negative regulation of cell proliferation
Adenomatous polyposis coli	182397	0.00010	10.1	0.6	0.12	cell migration and adhesion, transcriptional activation and apoptosis
TAR DNA-binding protein 43	6678271	0.00020	10.2	4.3	0.82	DNA/RNA binding protein
heat shock protein 70kDa 1A/B	167466173	0.00020	10.2	2.3	0.92	molecular chaperone
hCG1816057	119591190	0.00050	10.1	22.6	0.12	unknown
PDPK1 3-phosphoinositide dependent protein kinase-1	47680169	0.00050	10.1	6.3	0.03	T cell receptor signaling pathway, activation of protein kinase B focal adhesion assembly, phosphatidylinositol-mediated signaling phosphorylation, platelet activation, regulation of I-kappaB kinase/NF-kappaB cascade
nucleoporin 205 (NUP205)	1504030	0.00060	10.1	0.7	0.01	Ubiquitin ligase substrate identification through quantitative proteomics at both the protein and peptide levels.
suppressor of tumorgeniity 14 protein	11415040	0.00070	10.1	2.5	0.00	systematic and quantitative assessment of the ubiquitin-modified proteome.
MIP18 familiy protein FAM96A isoform a precursor	14149934	0.00070	10.1	8.8	0.02	Systematic and quantitative assessment of the ubiquitin-modified proteome.
fibrocystin isoform 1 precursor	126131102	0.00080	10.1	0.3	0.00	Kidney development, Calcium inon homeostasis, regulator of ERK1 and 2, NF-kappaB, protein kinase B signaling
unnamed protein product	47077803	0.00080	10.1	7.4	0.08	unknown
BTB/POZ zinc finger protein DPZF	13386602	0.00080	10.1	3	0.01	DNA binding, leuci zipper, metal binding

Recombinant TDP-43-FLAG proteins were incubated with reaction buffer containing all components for in vitro ubiquitination. After adding disulfide cross-linker, dithiobis[succinimidyl propionate] (DSP), reaction mixture was incubated with anti-FLAG affinity beads. After releasing from TDP-43 under the mild reducing conditions, protein pulled-down with TDP-43 were trypsinized and analyzed by LC-MS/MS. The acquired MS/MS spectra were automatically searched against the protein database in NCBI using the TurboSEQUEST. P, peptide probability. Sf, Sf-Final score.

Supplementary Table S2

Cloning primer pairs		
pcDNA3-TDP-43-FLAG	forward	5’-TGCGCAGTCTCTTTGTGGACAGGACTTGATCATTA-3’
E246Q	reverse	5’-TAATGATCCTGTCCACAAAGAGACTGCGCA-3’
pcDNA3-TDP-43-FLAG	forward	5'-CGCAGTCTCTTTGTGGAGAGAACTTGATCATTAAAGGAATC-3’
D247N	reverse	5'-GATTCCTTTAATGATCAAGTTCTCTCCACAAAGAGACTGCG-3’
pcDNA3-TDP-43-FLAG	forward	5'-TGATCAGTGTGGAGAGGACTTGAT-3’
ΔNES1	reverse	5'-TCTCCACACTGATCATCTGCAAATG-3’
pcDNA3-TDP-43-FLAG	forward	5'-TGGAGAGAAAGGAATCAGCGTTCAT-3’
ΔNES2	reverse	5'-ATTCCTTTCTCTCCACAAAGAGACTG-3’
pcDNA3-TDP-43-FLAG	forward	5'-TACCGAGCTCGGATCCAAGATGGCTTCATCAGCAG-3’
CTF35	reverse	5'-TAGATGCATGCTCGAGTCACTTGTCGTCATCGTCT-3’
pcDNA3-Myc-CUL2	forward	5'-GGGGGATCCGTTCTTTGAAACCAAGAGTAGTAG-3’
	reverse	5'-CCCGGTACCTCACGCGACGTAC|GCTGTATT-3’
pcDNA3-HA-VHL	forward	5'-GGGAAGCTTGATGCCCCGGAGGGCGGAGAACTGGGA-3’
	reverse	5'-CCCGAATTCAATCTCCCATCCGTTGAT-3’
siRNA		
CUL2_#1	forward	5'-GGUGCAGACUAUAUGGACUGCUUAU-3’
	reverse	5'-AUAAGCAGUCCAUAUAGUCUGCACC-3’
CUL2_#2	forward	5'-GGUAUCUCAACACCCAGUUUAUUAA-3’
	reverse	5'-UUAAUAAACUGGGUGUUUGAGAUACC-3’
CUL2_#3	forward	5'-CAGAGAACCUAAGUCUGUUUGCAAA-3′
	reverse	5'- UUUGCAAACAGACUUAGGUUCUCUG-3′ .
TDP-43_#1	forward	5'-AAGACUUAGAAUCCAUGCUUGAGCC-3’
	reverse	5'-GGCUCAAGCAUGGAUUCUAAGUCUU-3’
TDP-43_#2	forward	5'-UUUCACUGCUGAUGAAGCAUCUGUC-3’
	reverse	5'-GACAGAUGCUUCAUCAGCAGUGAAA-3’
TDP-43_#3	forward	5'-UGAAUGACCAGUCUUAAGAUCUUUC-3’
	reverse	5'-GAAAGAUCUUAAGACUGGUCAUUCA-3’
real time-PCR		
VHL	forward	5'-TACCGAGGTCACCTTTGGCTC-3’
	reverse	5'-TCTCCCATCCGTTGATGTG-3’
GAPDH	forward	5'-GCACCGTCSSGGCTGAGAAC-3’
	reverse	5'-TGGTGGTGAAGACGCCAGTGGA -3’

Sequence information of oligonucleotides for cloning, siRNA, and real-time PCR

Supplementary Table S3

Antigen (clone)	Company	origin	ICC	IHC	IB
FLAG (M2)	SIGMA	mouse	1:500	N.A.	1:500
HA (3F10)	Roche	rat	1:300	N.A.	1:500
GFP	Nacalai tesque	rat	N.A.	N.A.	1:500
TARDBP	Proteintech	rabbit	1:500	1:500	1:500
CUL2	Abcam	rabbit	1:200	N.A.	1:500
VHL	Thermo-Fischer	rabbit	N.A.	N.A.	1:500
ElonginB	Santa Cruz	rabbit	N.A.	N.A.	1:500
ElonginC	Santa Cruz	rabbit	N.A.	N.A.	1:500
ubiquitin K48	Milipore	rabbit	1:500	N.A.	1:500
Hsc70/Hsp70	StressGen	rabbit	1:200	N.A.	1:500
phospho-TDP-43	Cosmobio	rabbit	N.A.	1:300	N.A.
GST-pi	MBL	rabbit	N.A.	0.1805556	N.A.
PDGFRα	Santa Cruz	rabbit	N.A.	N.A.	1:500
MBP	MBL International	rabbit	N.A.	N.A.	
GAPDH	Santa Cruz	rabbit	N.A.	N.A.	1:500
actin	Santa Cruz	mouse	N.A.	N.A.	1:1000
VHL	self-made	mouse	1:500	1:100	1:400
TDP-43(3B12A)	self-made	mouse	1:500	N.A.	N.A.
MBP; myelin basic protein; GST-pi; glutahione S-trensferase -pi					
PDGFRα, Platelet-Derived Growth Factor Receptor, Alpha Polypeptide					
ICC, immunocytochemistry; IHC immunohistochemistry; IB, immunoblotting.					
N.A. indicates "not assessed"					

Antibody information used in this work. ICC, immunocytochemistry; IHC immunohistochemistry; IB, immunoblotting. N.A. indicates "not assessed".

Supplementary Figure S1

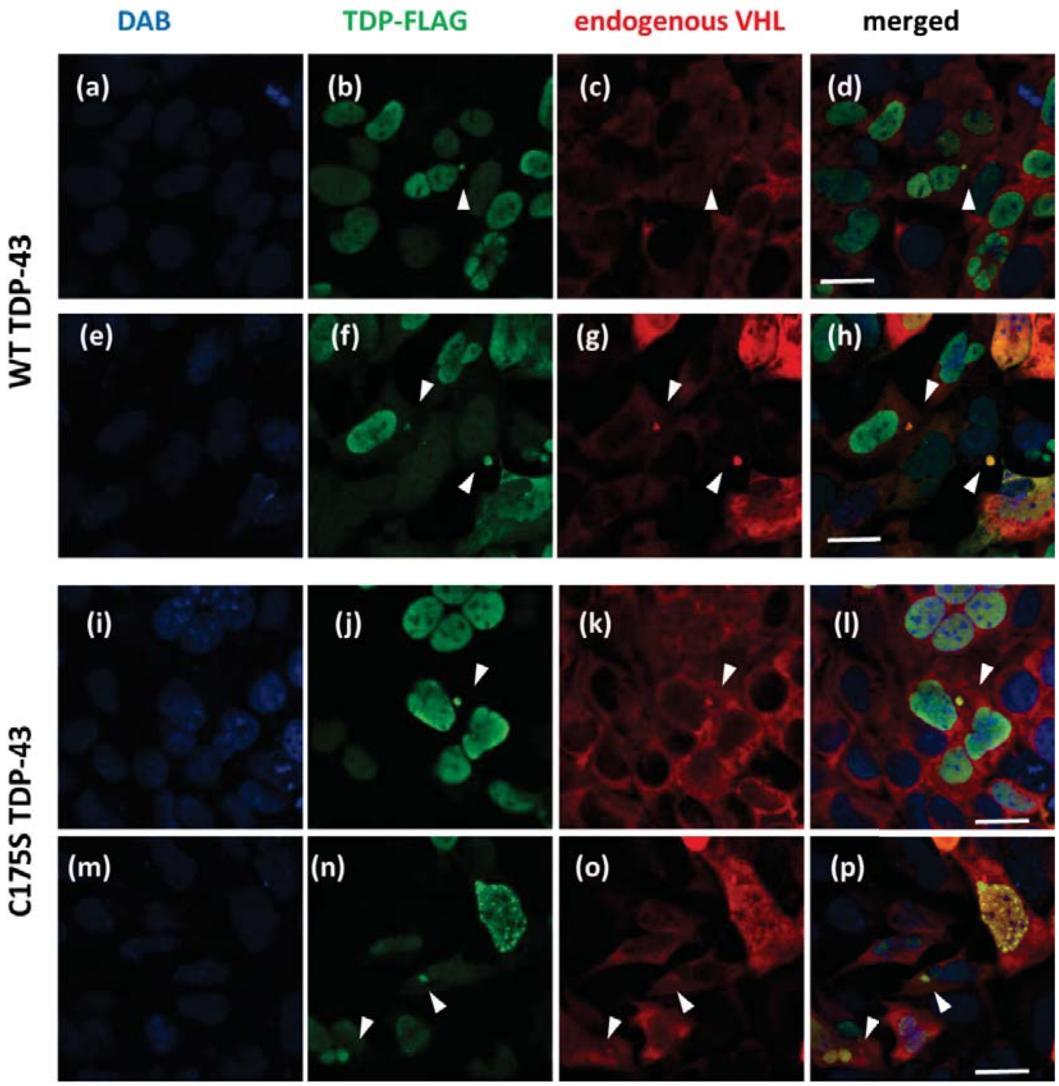

Confocal laser microscope analysis showing the colocalization of WT TDP-43 and endogenous VHL. HEK293A cells were transiently transfected with TDP-43-FLAG with (e-h, m-p) or without (a-d, i-l) HA-VHL. After 48 h, cells were fixed and immunostained for FLAG (green) and VHL (red). Arrowheads indicates perinuclear inclusions of WT TDP-43, which are also immunoreactive to VHL. Scale bar = 20 μm.

Supplementary Figure S2

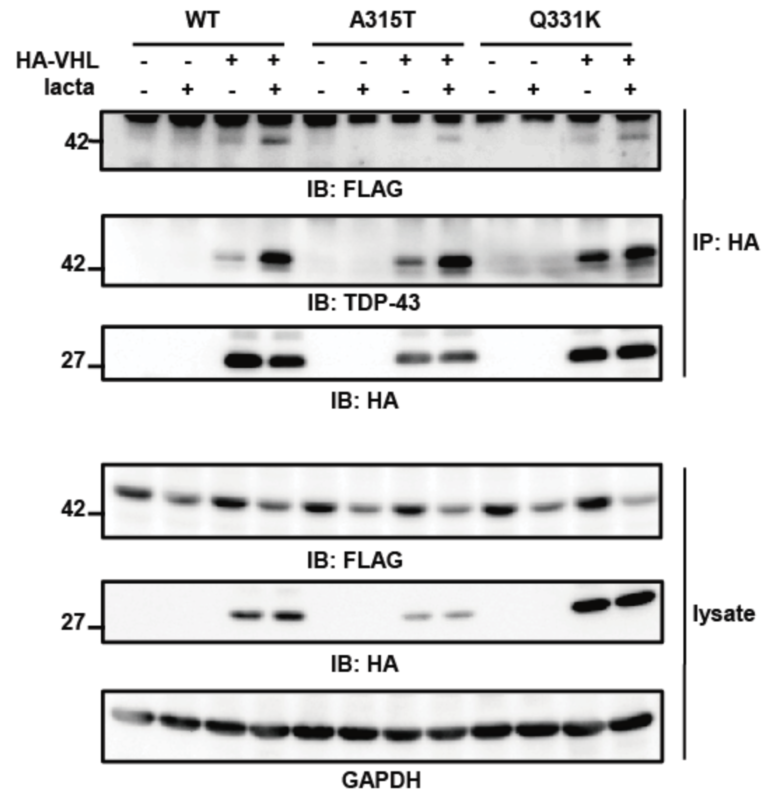

The binding affinity of FALS-linked TDP-43 with VHL. HeLa cells were transiently transfected with TDP-43-FLAG (WT, A315T, G331K) and HA-VHL. Cell lysates harvested 48 h after transfection were immunoprecipitated with anti-HA antibody, and analyzed by Western blotting for TDP-43 or FLAG. Note that FALS-linked TDP-43 displayed comparative binding with VHL.

Supplementary Figure S3

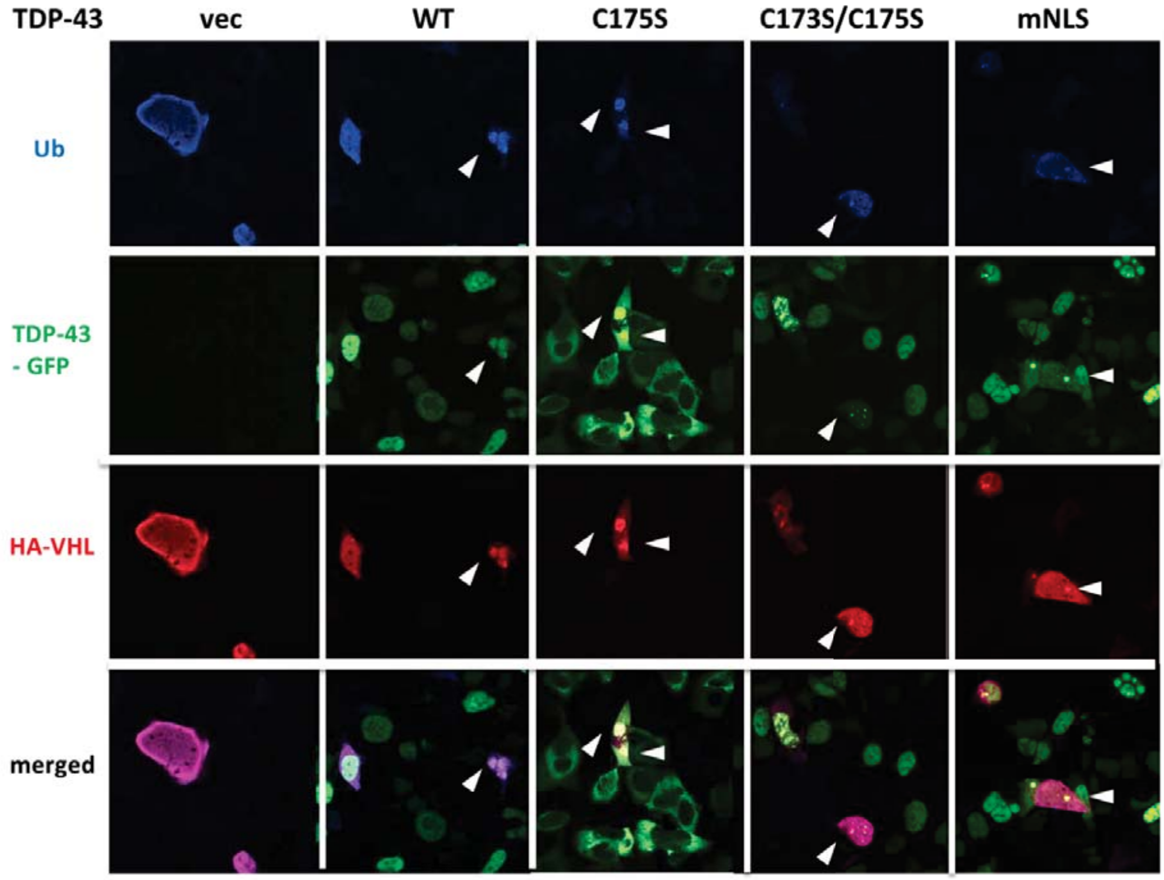

Confocal laser microscope analysis showing the colocalization pattern of WT or mutant TDP-43, ubiquitin, and HA-VHL. HEK293A cells were transiently transfected with TDP-43-EGFP (WT, C175S, C173S/C175S, mNLS) or GFP vector control and HA-VHL. After 48 h, cells were fixed and immunostained for ubiquitin K48 (blue) and HA (red). Arrowheads indicates perinuclear inclusions of TDP-43 and ubiquitin, which are also immunoreactive to overexpressed VHL. Scale bar = 20 μm.

Supplementary Figure S4

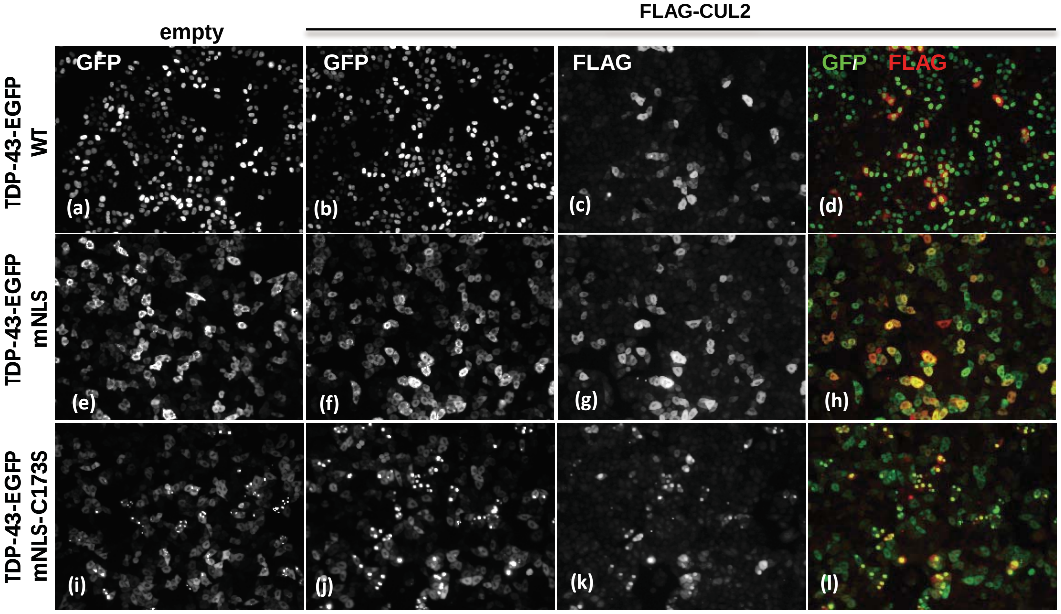

CUL2 colocalizes with aggregated TDP-43. Fluorescence microscope of HeLa cells expressing FLAG-CUL2 and TDP-43-EGFP of WT (a-e), mNLS (e-f), and double mutants with defective NLS and with substitutions at Cys173S for Ser (mNLS-C173S, i-l). Cells were fixed and immunostained for FLAG (red).

Supplementary Figure S5

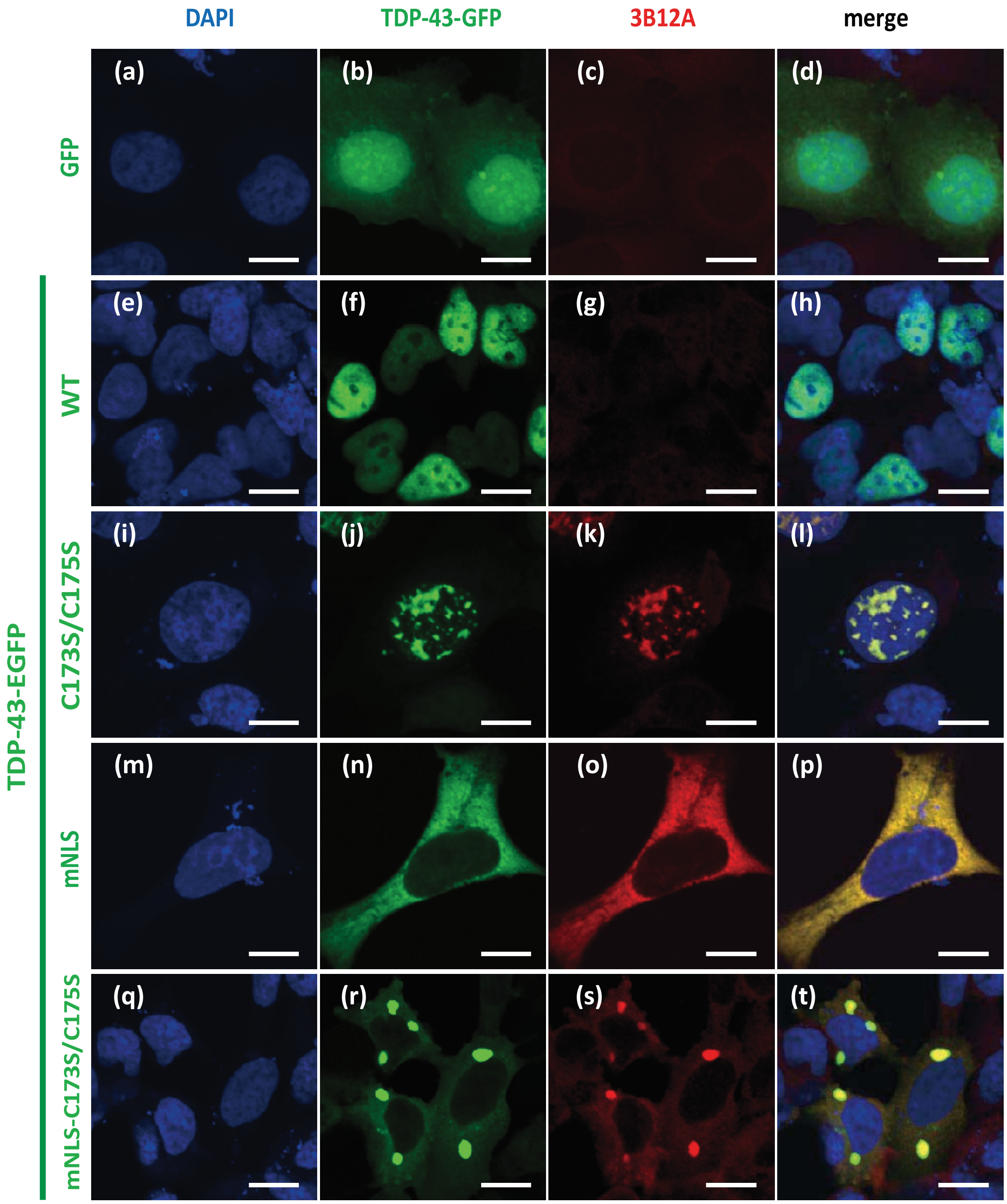

Confocal microscope analysis showing the E246/D247 is exposed in the aggregate-prone TDP-43. TDP-43. HEK293A cells were transfected with WT or C173S/C175S mutant of TDP-43-EGFP with or without mutant nucleus localizing signal (NLS). After 48 h, cells were fixed and were immunostained for 3B12A MAb,

raised against E246/D246 as a core epitope. Note that 3B12A recognizes only milocaized (m-p), aggregate-prone TDP-43 eiher in nucleus (i-l) or cytosol (q-t), but not WT TDP-43 (e-h) or EGFP (a-d) proteins. Scale bar=10 μm.

Supplementary Figure S6

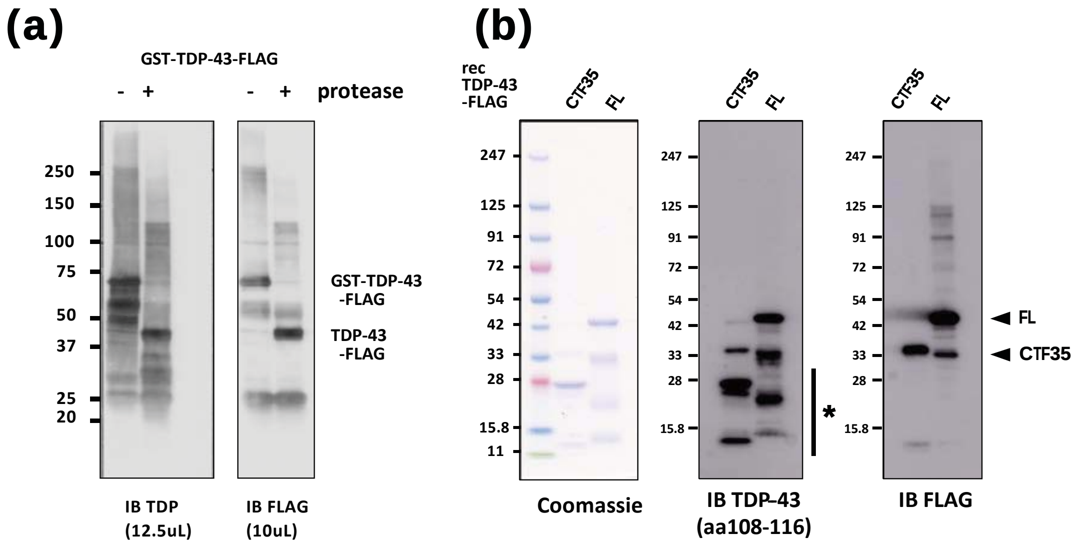

TDP-43 is mechanically fragile and readily fragmented *in vitro*. A. GST-tagged full-length TDP-43-FLAG proteins were induced by IPTG in E coli, and GST was released from TDP-43 by protease. a. The recombinant TDP-43 before (left panels) and after (right panels) protease treatment was analyzed with Western blotting, using antibodies against TDP-43 and FLAG. b. Recombinant FL TDP-43-FLAG and CTF35-FLAG, being stored at -80°C, immediately after protease cleavage and buffer replacement, were separated onto polyacrylamide gel, and were visualized with Coomassie brilliant blue (CBB), anti-TDP-43, and anti-FLAG antibodies. Note that smaller fragments, containing N-sided (by ant-TDP-43(108-116) and C terminal were ones, were abundantly detected, even in the carefully frozen condition.

Supplementary Figure S7

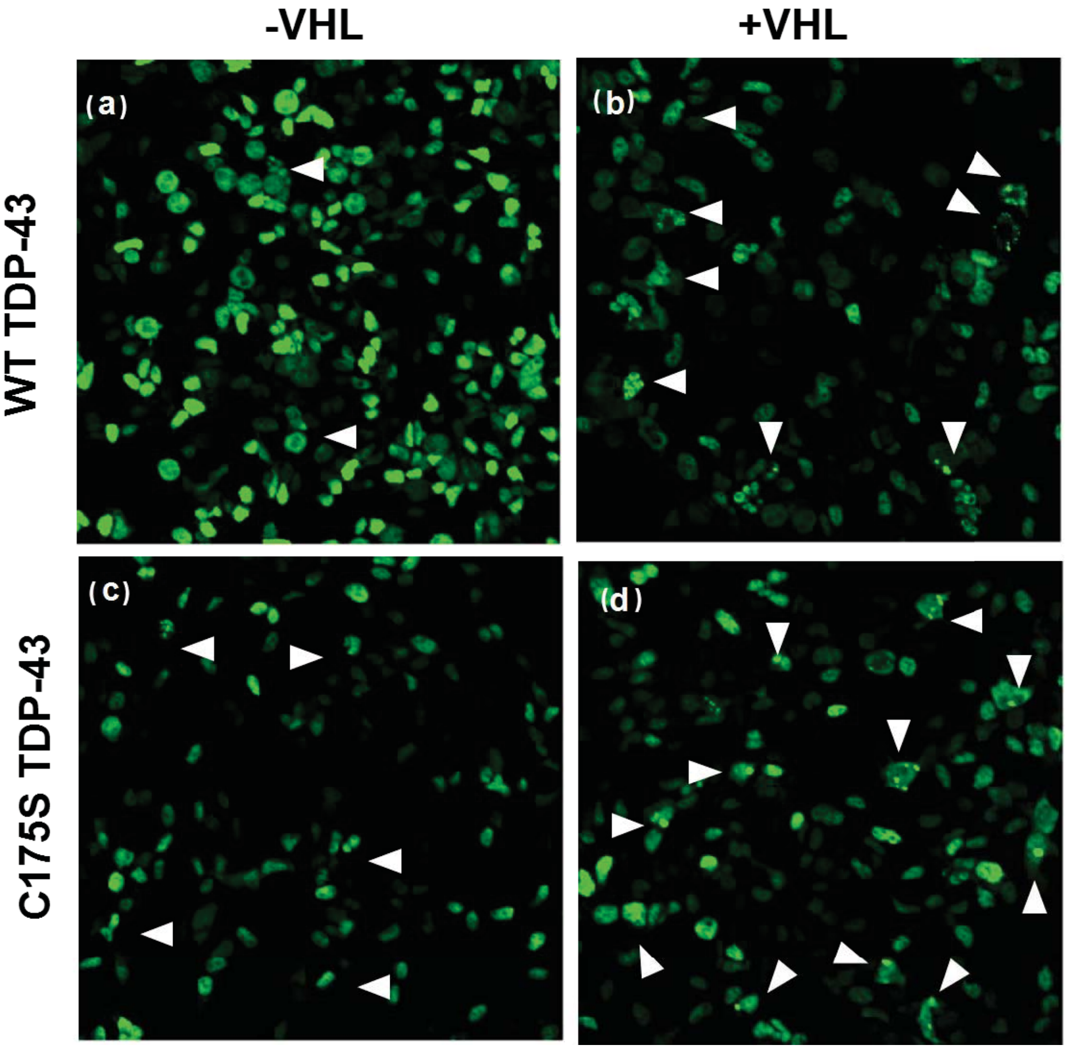

Effect of VHL overexpression on the aggregate formation of TDP-43 in HEK293A cells. HEK293A cells were transiently co-transfected with TDP-43-EGFP ((a) and (b) for WT, (c) and (d) for C175S) and HA-VHL or vector control. Forty-eight h after the transfection, cells were fixed with 4% PFA, and at least four photos containing more than 100 cells/image were randomly obtained, and analyzed unbiasedly, using image-J software. Arrows indicate counted cells with TDP-43 aggregates.

Supplementary Figure S8

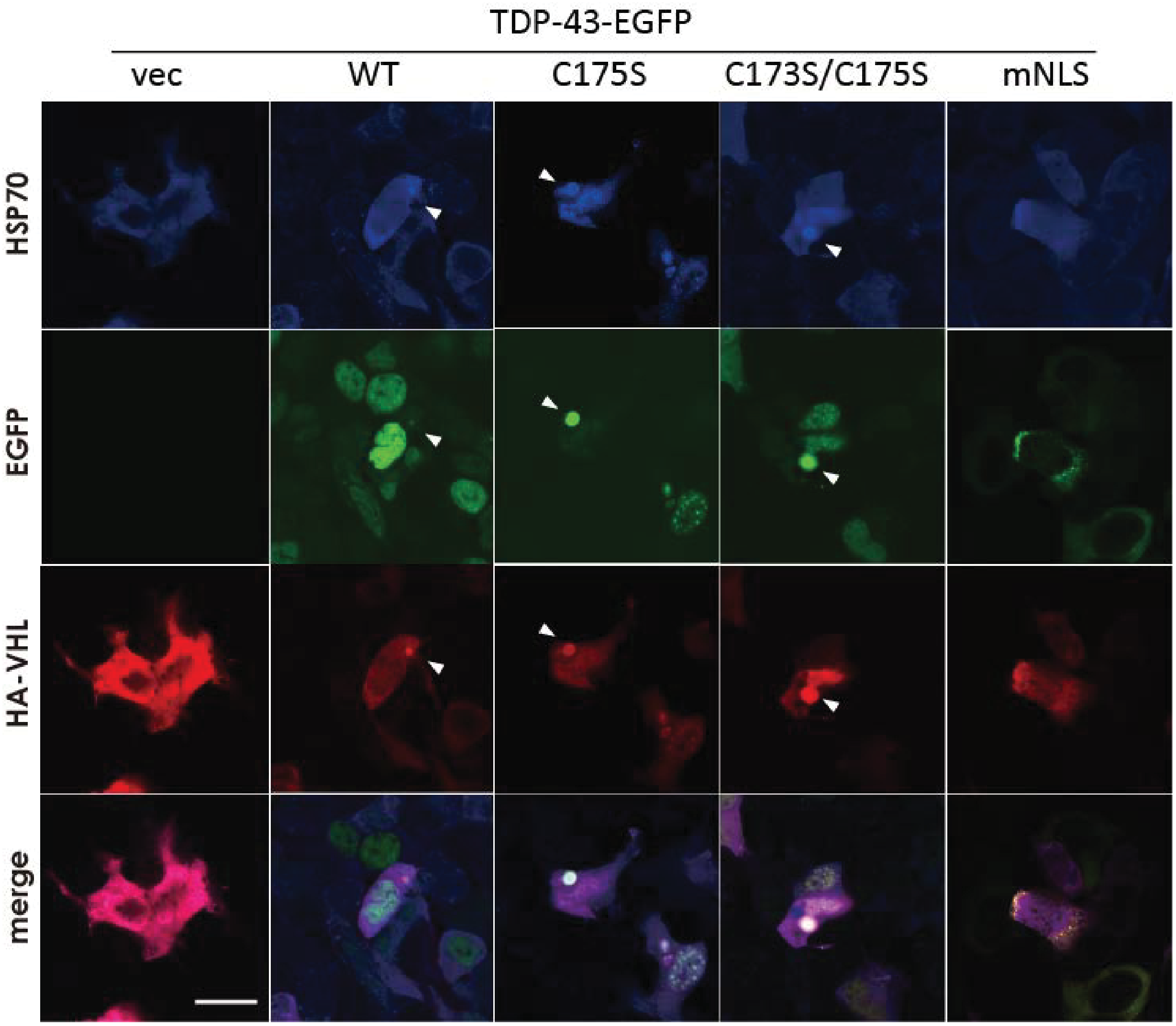

Confocal microscope analysis showing that TDP-43 inclusions co-localize with VHL and Hsp70.

HEK293A cells were transiently co-transfected with TDP-43-EGFP and HA-VHL. After 48 h, cells were fixed and immunostained for HA (red) and Hsp70 (blue). TDP-43-EGFP is indicated in green. Scale bar=10 μm.

Supplementary Figure S9

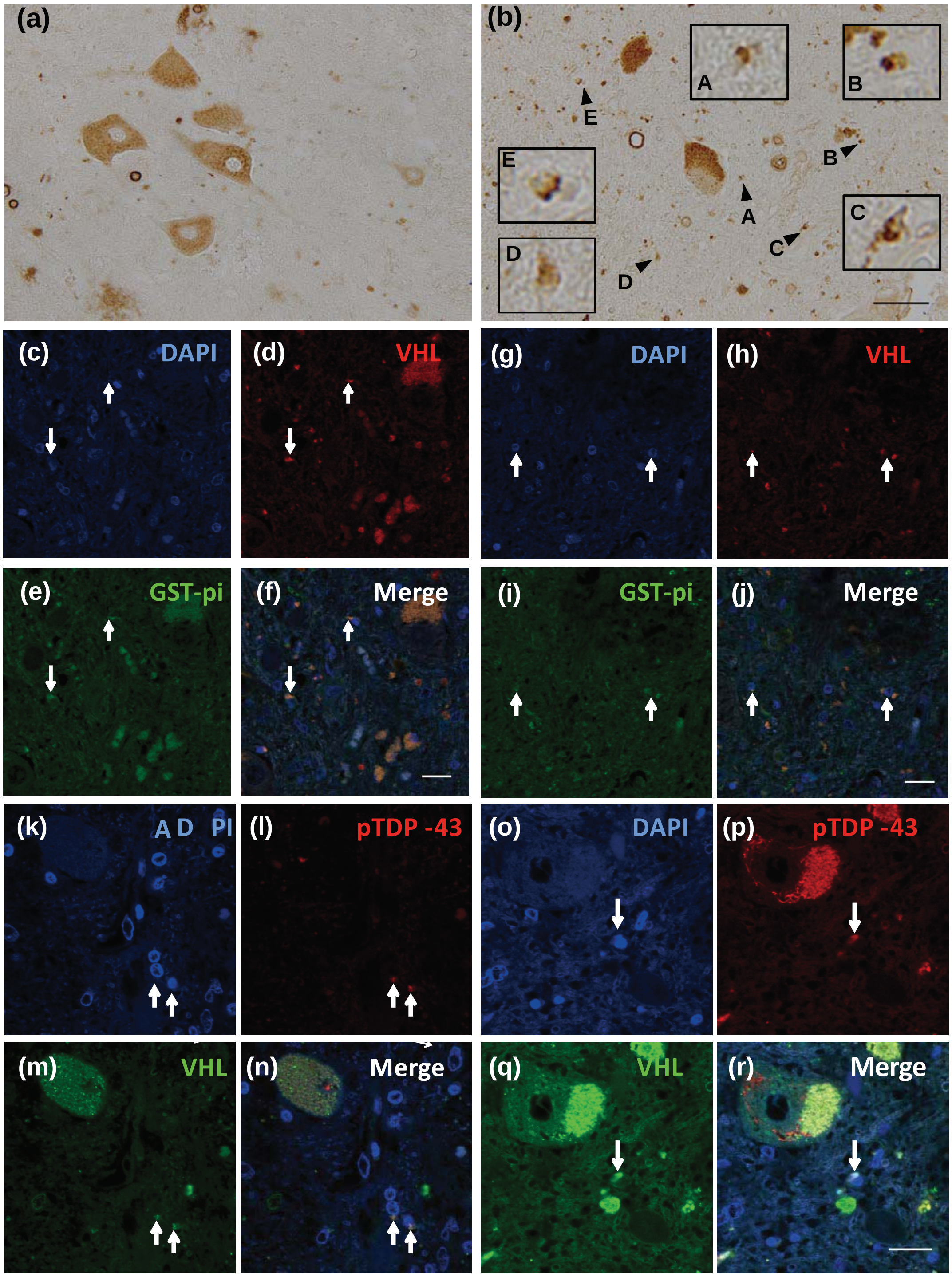

Colocalization of phosphorylated TDP-43 and VHL in the oligodendrocytes in ALS. Immunohistochemistry of spinal oligodendrocytes for VHL in the spinal cords from ALS patients (b-r) and ALS-irrelevant control subjects (a). In (b), cytoplasmic

aggregates of VHL in oligodendrocytes are indicated by arrowheads, glial cytoplasmic inclusions were magnified in the insets **(A-E), (c-r)** Double immunofluorescence study shows that the VHL colocalize with GST-pi (c-f, g-j), and with phosphorylated TDP-43 (k-n, o-r) in cytoplasmic inclusions in oligodendrocytes (arrows).Scale bar=50 μm.

Supplementary Figure S10

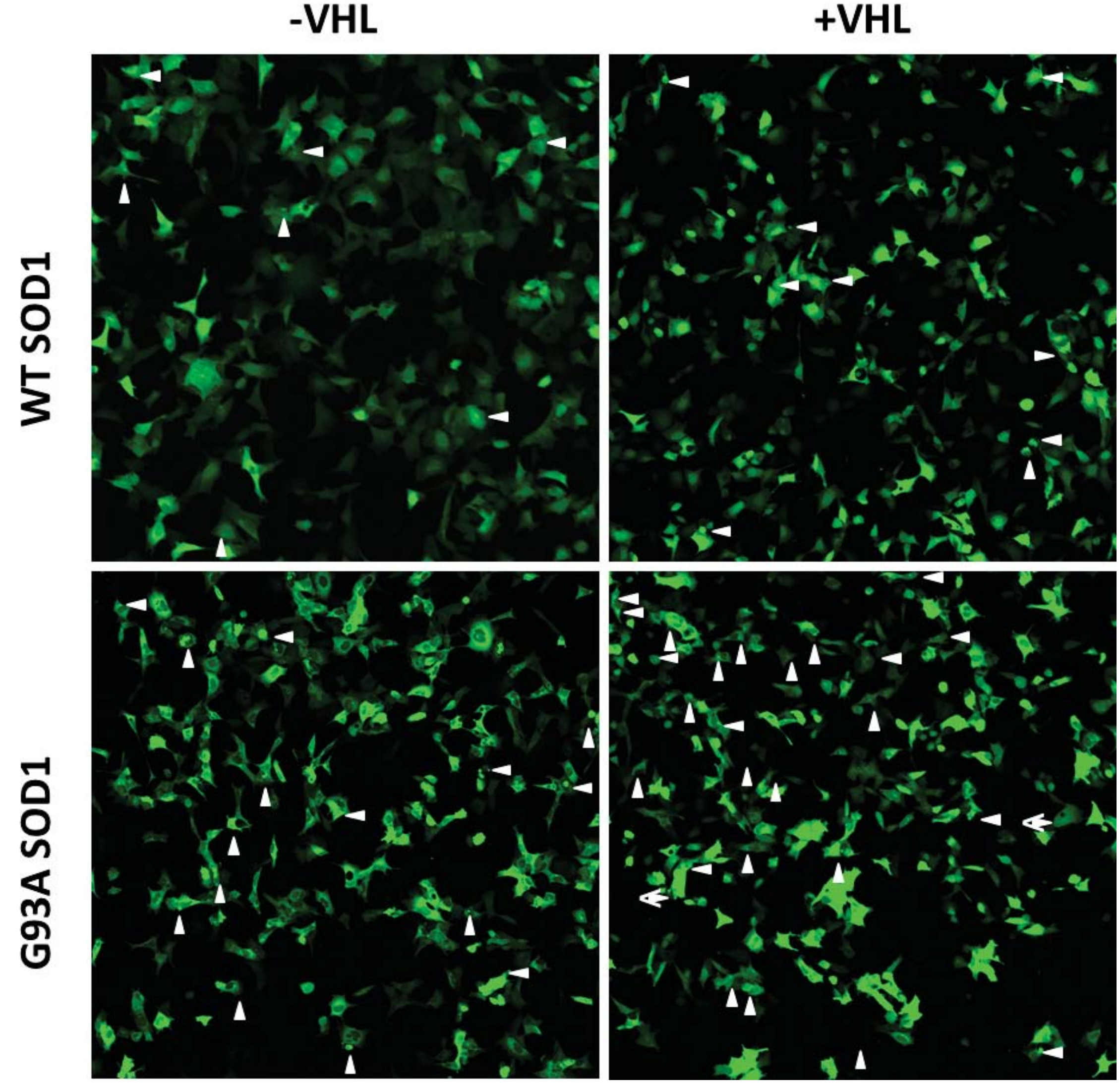

Effect of VHL overexpression on the aggregate formation of mutant SOD1 in HEK293A cells. HEK293A cells were transiently co-transfected with human SOD1-EGFP ((a) and (b) for WT, (c) and (d) for G93A) and HA-VHL or vector control. Forty-eight hr after the transfection, cells were fixed with 4% PFA, and at least four photos containing more than 50 cells/image were randomly obtained, and analyzed unbiasedly, using image-J software. Arrows indicate counted cells with SOD1 aggregates.

Supplementary Figure S11

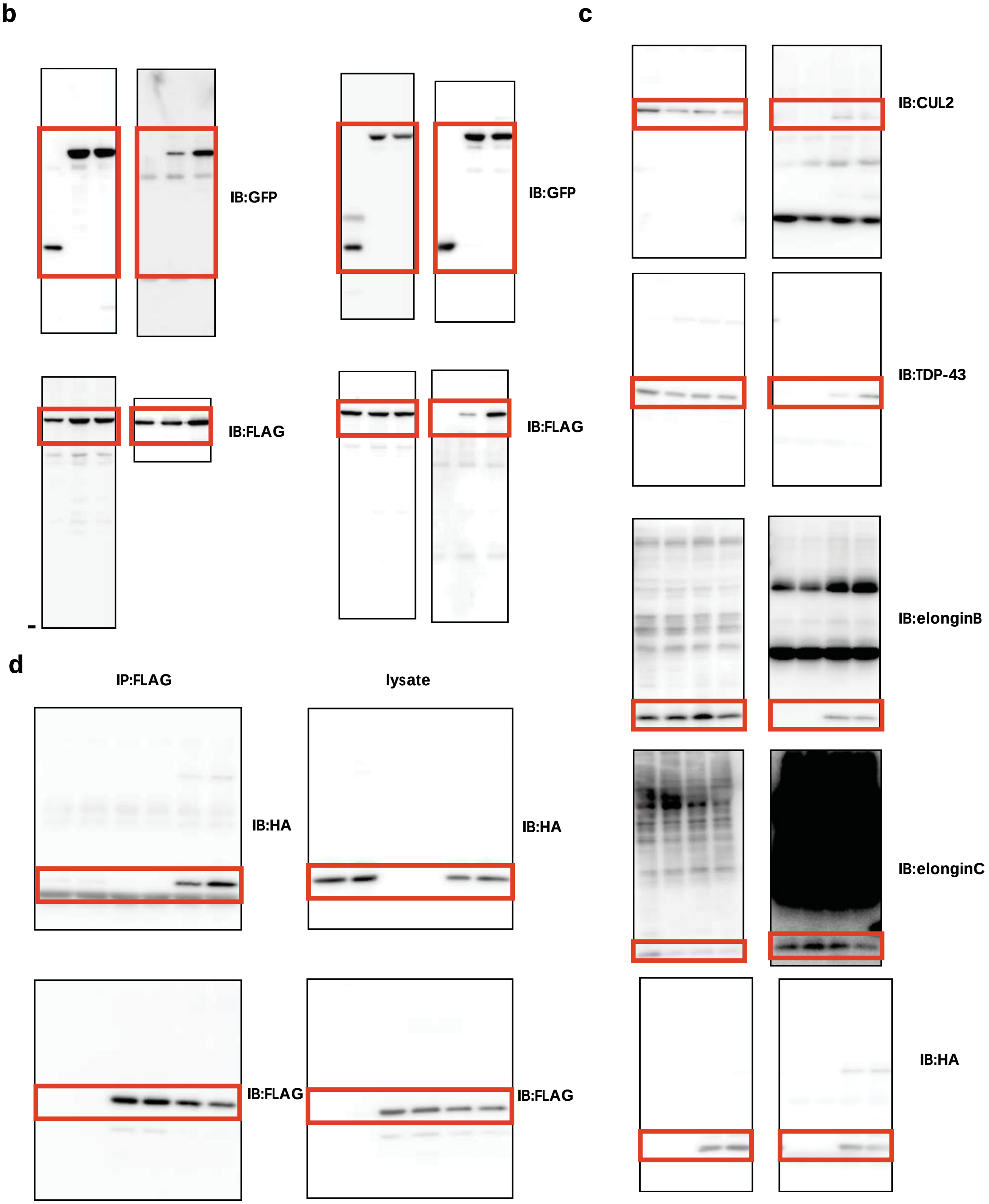

Original Western blots of trimmed panels in Fig. 1.

Supplementary Figure S12

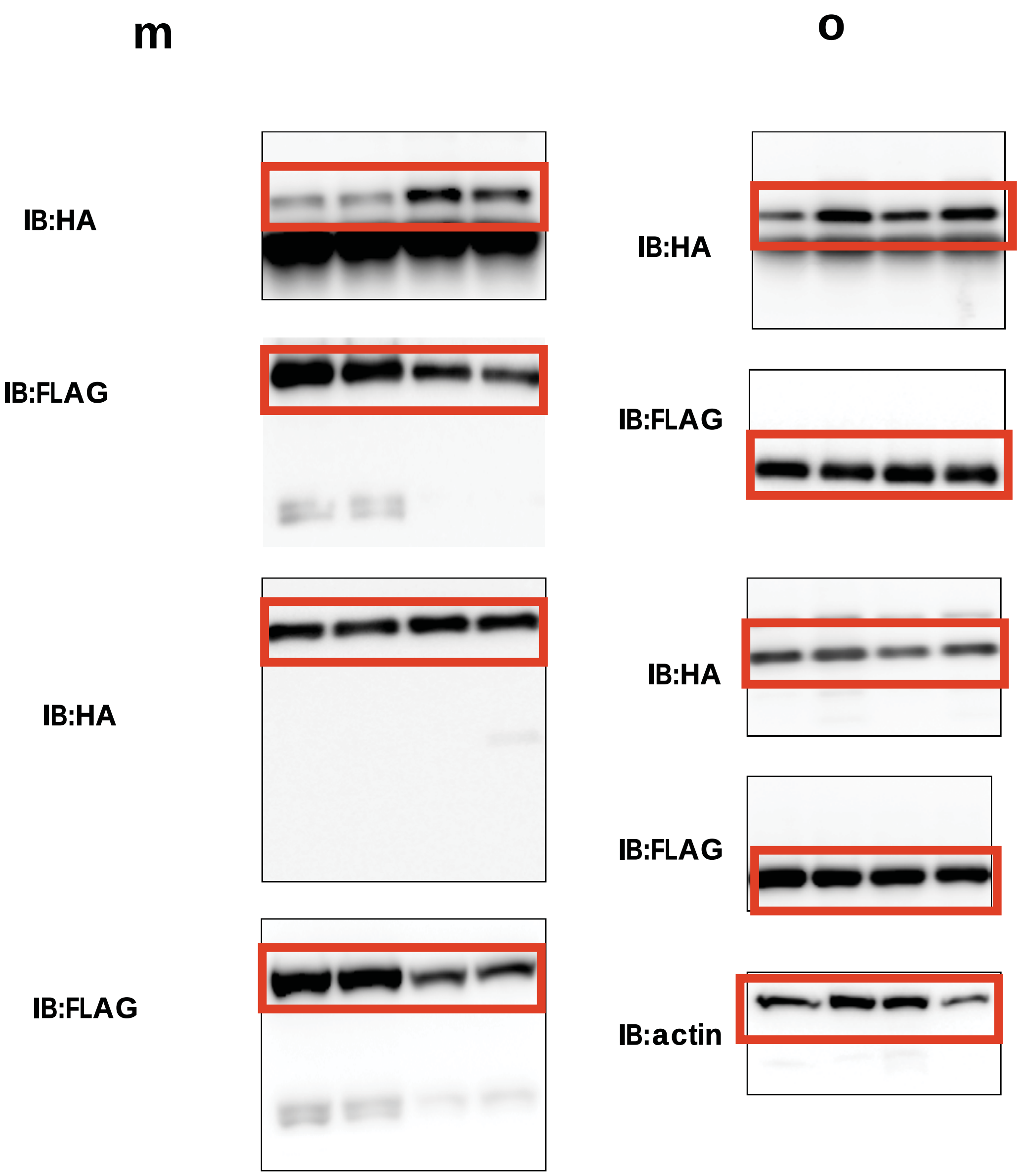

Original Western blots of trimmed panels in Fig. 2.

Supplementary Figure S13

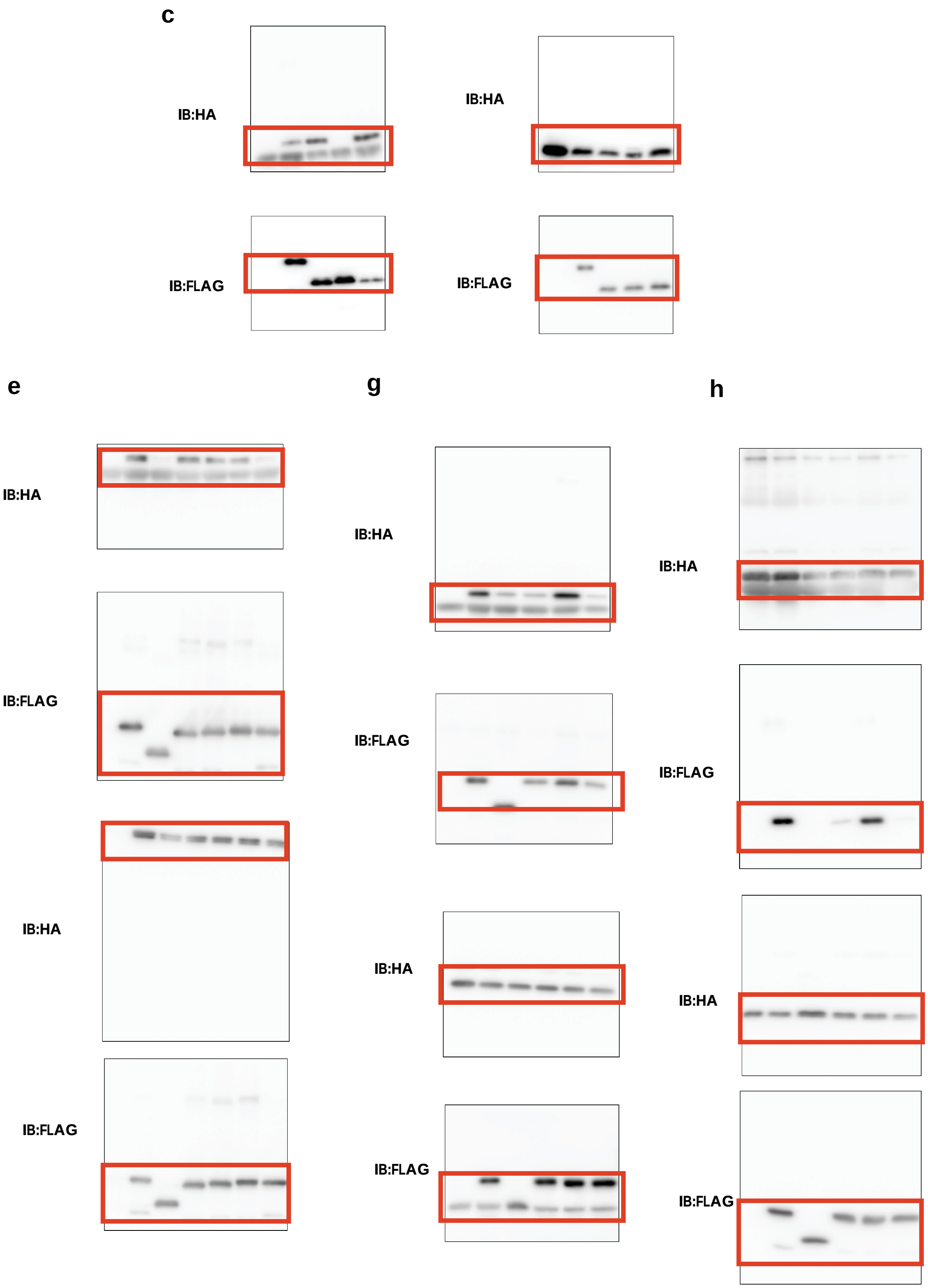

Original Western blots of trimmed panels in Fig. 3.

Supplementary Figure S14

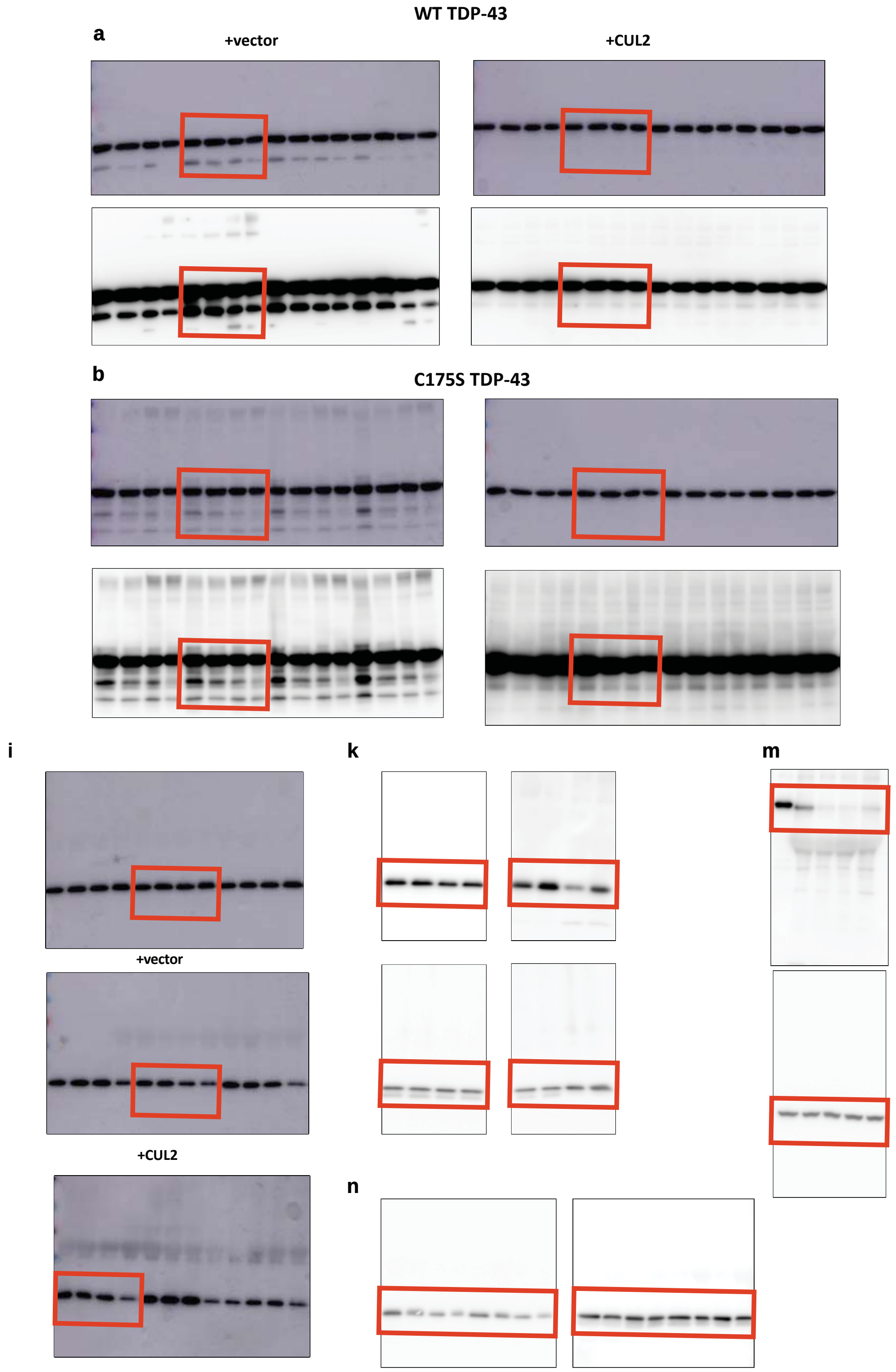

Original Western blots of trimmed panels in Fig. 4.

Supplementary Figure S15

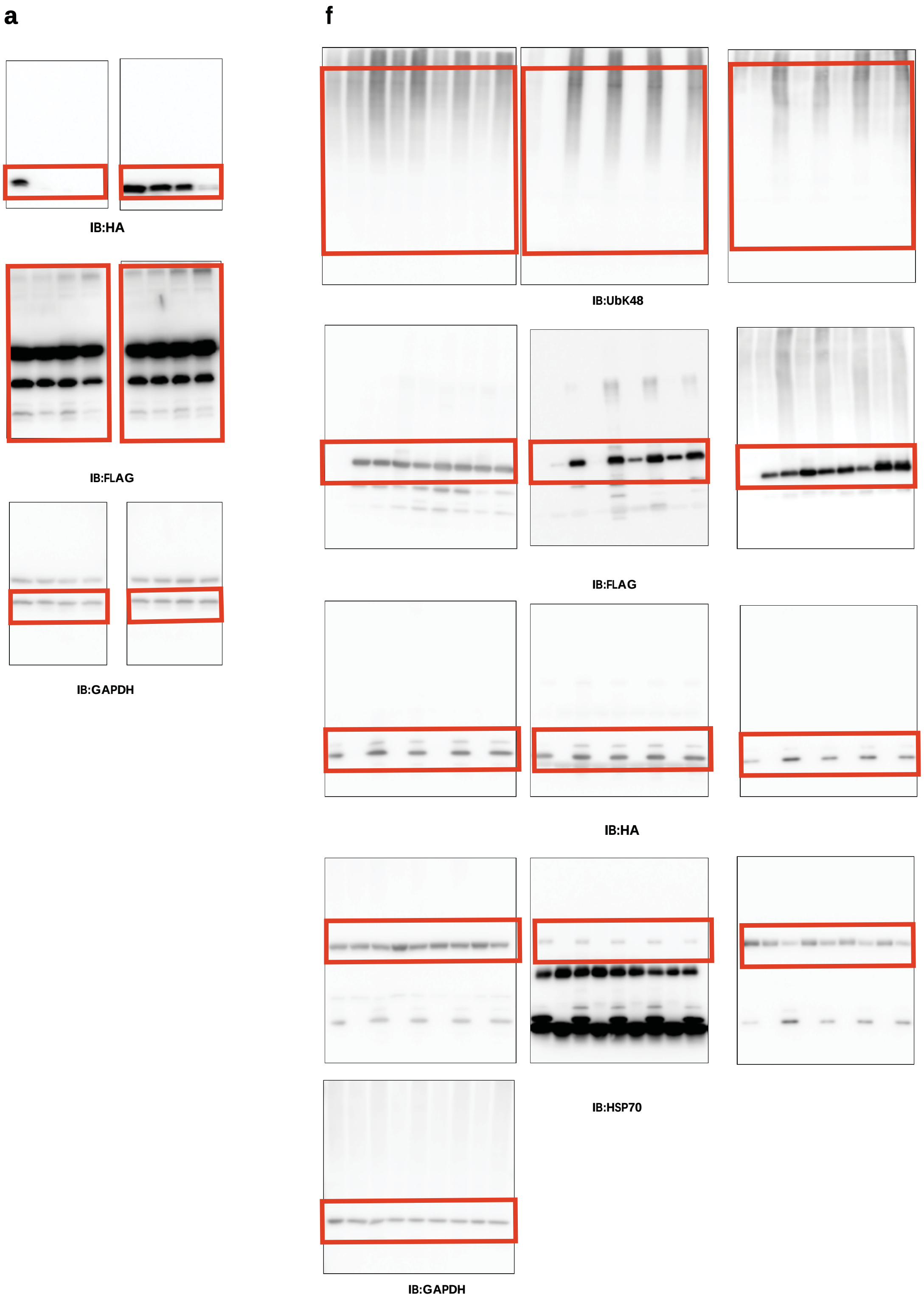

Original Western blots of trimmed panels in Fig. 7

Supplementary Figure S16

a

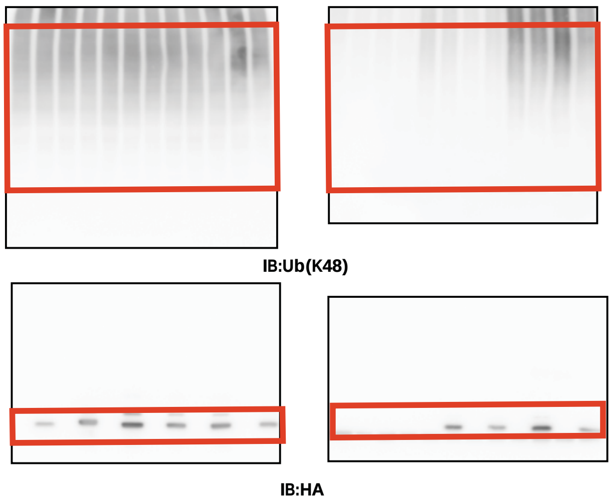

b

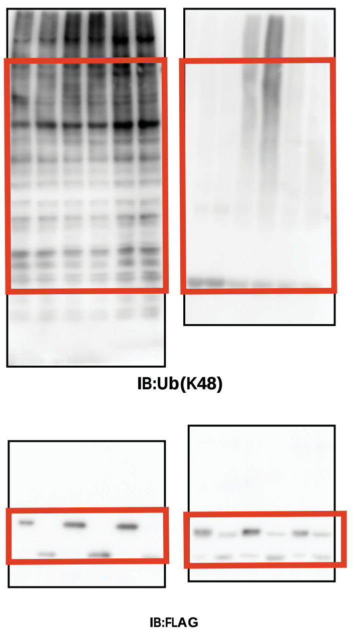

c

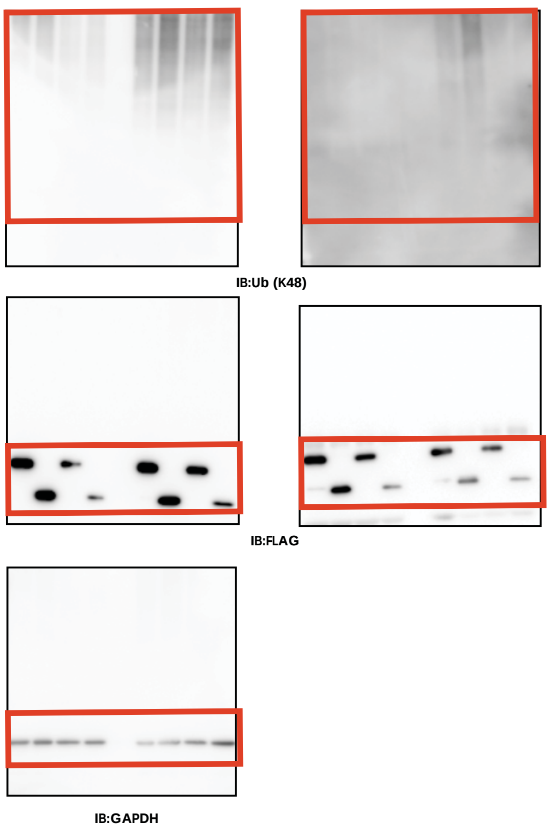

Original Western blots of trimmed panels in Fig. 5.

Supplementary Figure S17

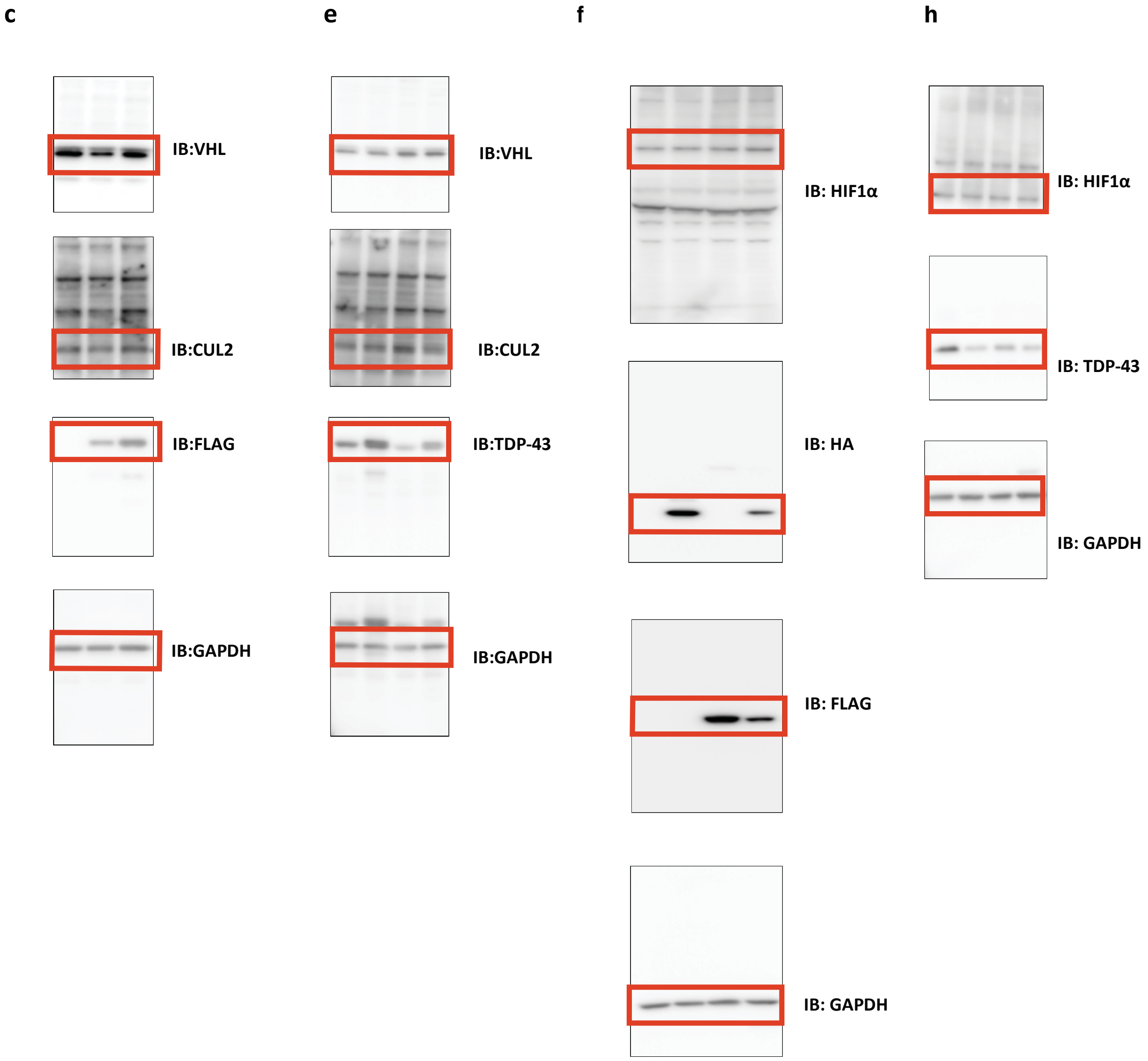

Original Western blots of trimmed panels in Fig. 8

Supplementary Figure S18
q

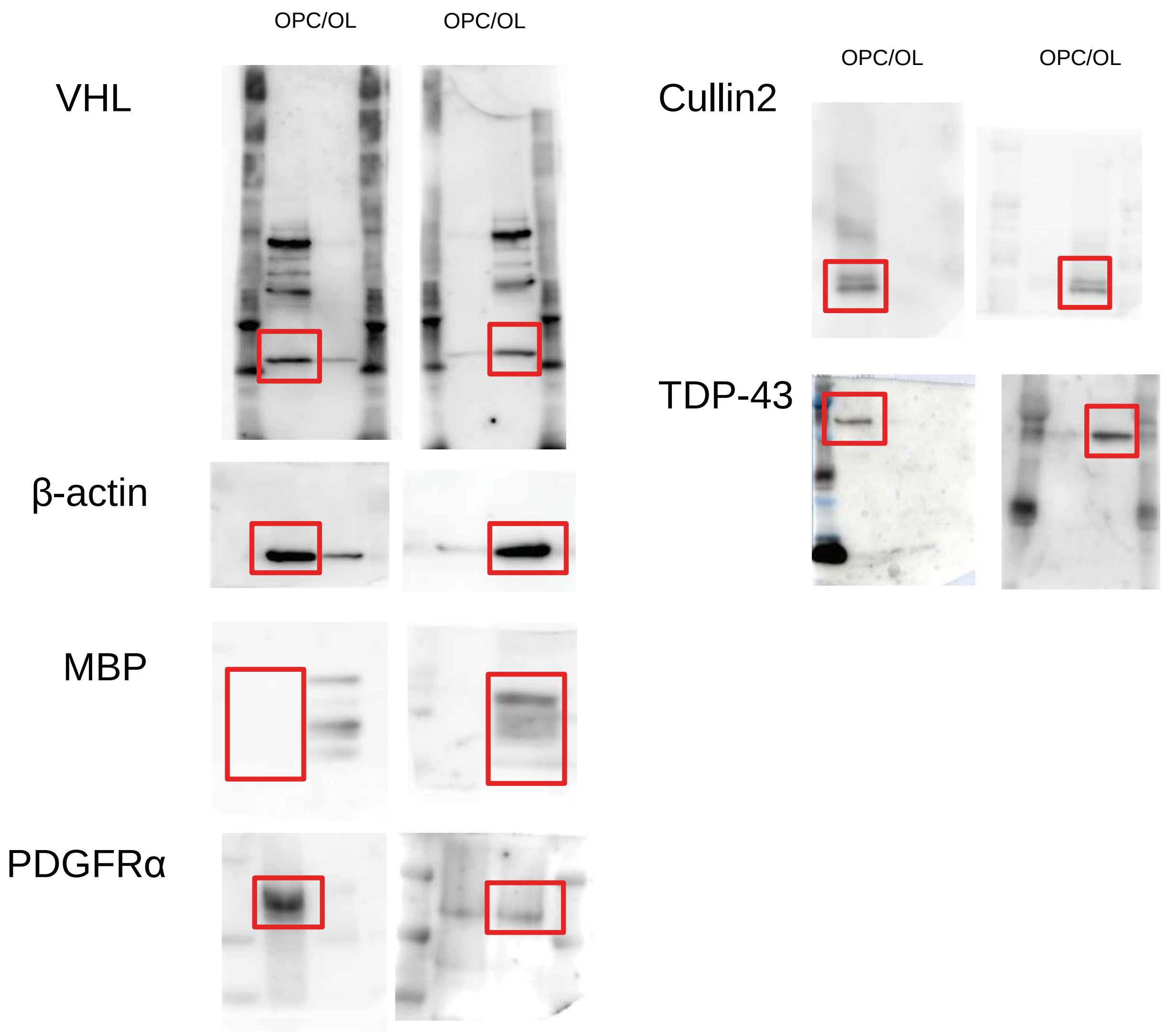

Original Western blots of trimmed panels in Fig. 9

Supplementary Figure S19

a

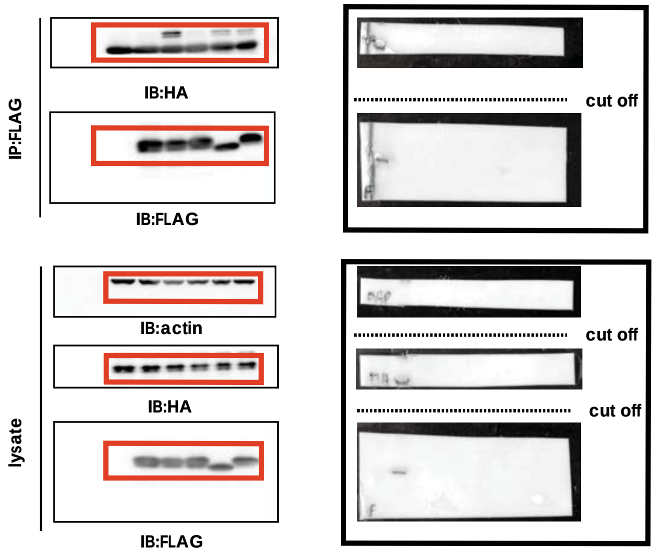

c

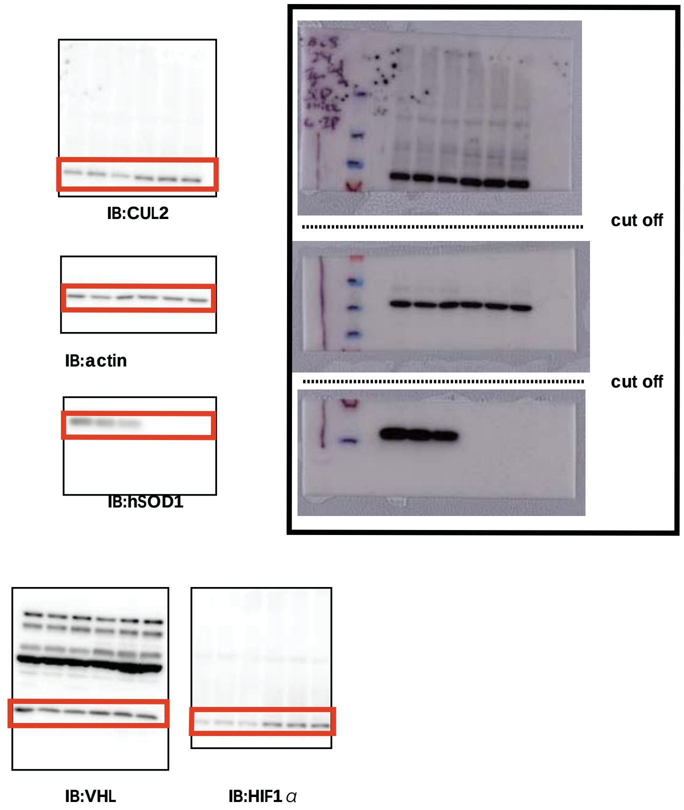

Original Western blots of trimmed panels in Fig. 10.
